# Comparison of factor analysis models applied to the NCANDA neuropsychological test battery

**DOI:** 10.1371/journal.pone.0263174

**Published:** 2022-02-10

**Authors:** Kevin M. Cummins, Eileen V. Pitpitan, Ty Brumback, Tyler M. Moore, Ryan S. Trim, Duncan B. Clark, Sandra A. Brown, Susan F. Tapert

**Affiliations:** 1 Department of Public Health, California State University, Fullerton, Fullerton, California, United States of America; 2 Joint Doctoral Program in Interdisciplinary Research on Substance Use, Division of Infectious Diseases and Global Public Health, Department of Medicine, University of California San Diego, La Jolla, California, United States of America; 3 School of Social Work, San Diego State University, San Diego, California, United States of America; 4 Department of Psychological Science, Northern Kentucky University, Highland Heights, Kentucky, United States of America; 5 Brain Behavior Laboratory, Department of Psychiatry, Perelman School of Medicine, University of Pennsylvania, Philadelphia, Pennsylvania, United States of America; 6 Department of Psychiatry, University of California San Diego, La Jolla, California, United States of America; 7 VA San Diego Healthcare System, San Diego, California, United States of America; 8 Department of Psychiatry, University of Pittsburgh, Pittsburgh, Pennsylvania, United States of America; 9 Department of Psychology, University of California San Diego, La Jolla, California, United States of America; University of Texas at Arlington, UNITED STATES

## Abstract

The factor structure of neuropsychological functioning among a large sample (N = 831) of American youth (ages 12–21 at baseline) was investigated in order to identify an optimal model. Candidate models were selected based on their potential to provide service to the study of adolescent development and the effects of heavy episodic alcohol consumption. Data on neuropsychological functioning were obtained from the NCANDA study. This is a longitudinal community study of the effects of alcohol exposure on neurodevelopment. Three conceptually motivated and one empirically motivated factor analysis model of neuropsychological domains were compared based on penalized-likelihood selection criteria and model fit statistics. Two conceptually-motivated models were found to have adequate fit and pattern invariance to function as a measurement model for the Penn Computerized Neurocognitive Battery (Penn CNB) anchored neuropsychological battery in NCANDA. Corroboration of previous factor analysis models was obtained, in addition to the identification of an alternative factor model that has higher discriminant capacity for neuropsychological domains hypothesized to be most sensitive to alcohol exposure in human adolescents. The findings support the use of a factor model developed originally for the Penn CNB and a model developed specifically for the NCANDA project. The NCANDA 8-Factor Model has conceptual and empirical advantages that were identified in the current and prior studies. These advantages are particularly valuable when applied in alcohol research settings.

## Introduction

Adolescence is an important period of neuromaturation and a sensitive period for persistent alterations to the brain’s reward circuitry resulting from alcohol exposure in animal models [1–3]. Emerging evidence suggests that patterns of alcohol exposure experienced by youth can result in alterations in neuroanatomical development [4], and associated neuropsychological deficits in many domains including, verbal learning [5–7], visuospatial processing [8], executive functioning [9], attention [8, 10] and memory [7, 9–11]. The functional implications of alcohol-induced neuromodulation are just now being confirmed in large-scale longitudinal community samples [12–14]. These studies are designed to provide a more definitive evaluation of adolescence as a sensitive period for the neurological influence of substance use exposure in humans.

The National Consortium on Alcohol and Neurodevelopment in Adolescence (NCANDA) is one of the large-scale studies designed to evaluate neuropsychological changes associated with alcohol use. In order to address its objectives, NCANDA’s protocol includes a neuropsychological test battery specifically designed to be sensitive to hypothesized decrements in neuropsychological functioning due to alcohol exposure. Cross-sectional patterns observed among conceptually-driven composite scores of neuropsychological tests have been reported for NCANDA [15]; however, evaluation of a factor structure in the NCANDA sample has not been evaluated.

The application of factor analysis to neuropsychological test batteries has been common. Accounts of human intelligence and cognitive architecture are anchored in factor analysis; for example, factor models described by Halstead [16], Newby et al. [17], and Patt et al. [18] have been influential to the field’s conceptual and methodological development. In addition to providing insight into cognitive architecture, factor analysis has been employed to achieve the statistical benefits of dimension reduction and reduced estimation error [19]. Factor analysis has been applied to the Penn CNB [20], which comprises the core components of the NCANDA test battery [15].

The factor structure of the Penn CNB tests in a large sample of youth (age range 8–21) was found to be similar to the proposed conceptual organization (Gur Model) used to develop this standardized battery [20]. The single exception was between the conceptual model and the empirically-based model determined through exploratory factor analysis was that the Penn CNB’s conditional exclusion test designed to measure abstraction and mental flexibility. In the factor analysis, conditional exclusion loaded onto the Complex Cognition latent factor based on efficiency scores, as opposed to the presumed Executive Functioning factor [20]. This empirically derived factor model reported by Moore et al. [20] is referred to as the Empirical Model in this paper.

Sullivan et al. [15] proposed an organization of the NCANDA test battery, which includes twelve tasks from the Penn CNB and six additional tasks from other test batteries (Table 1). The eight functional domains proposed by Sullivan et al. [15] were based on the conceptual neuropsychological domains from the tests’ initial development. Thus, Sullivan and colleagues split executive functioning into single task indices of attention (Continuous Performance Test) and working memory (N-back Task) and grouped the Conditional Exclusion Task with other tests of abstraction rather than executive functioning (Table 1). This organization of neuropsychological tests uncovered better performance among participants with limited alcohol exposure compared to those with moderate alcohol exposure in the balance domain and in five speed domains [15].

**Table 1:** Configural models evaluated in this study.

Assessment		Domain	Suite	8-Factor	Vulnerabilities†	Gur+	Empirical
	References			[15]	[3]	[20]	[20]
Penn Continuous Performance Test	[23]	Attention	1,2	Attention	Attention	Executive Functioning	Factor 1
N-Back Task	[23]	Working Memory	1,2	Working Memory	Attention	Executive Functioning	Factor 1
Penn Face Memory task	[23]	Face Memory	1,2	Episodic Memory	Memory	Episodic Memory	Factor 2
Penn Delayed Face Memory	[24]	Face Memory	1,2	Episodic Memory	Memory	Episodic Memory	
Visual Object Learning Test	[23]	Spatial Memory	1,2	Episodic Memory	Memory	Episodic Memory	Factor 1 & 2
Delayed Visual Object Learning Test	[23]	Spatial Memory	1,2	Episodic Memory	Memory	Episodic Memory	
Penn Word Memory Task	[23]	Verbal Memory	1,2	Episodic Memory	Verbal Learning	Episodic Memory	Factor 2
Penn Continuous Exclusion Test	[23]	Mental Flexibility	1,2	Abstraction	Visuospatial Processing	Executive Functioning	Factor 1
Penn Verbal Reasoning Test	[23]	Language Reasoning	1,2	Abstraction	Verbal Learning	Complex Cognition	Factor 1
Penn Matrix Reasoning Test (Special FORM A)*	[23]	Nonverbal Reasoning	1,2	Abstraction	Visuospatial Processing	Complex Cognition	Factor 1
Penn Emotion Differentiation Test	[23]	Emotion Differentiation	1,	Social Cognition	Social Cognition	Social Cognition	Factor 3
Penn Emotion Recognition Test	[23]	Emotion Identification	1,2	Social Cognition	Social Cognition	Social Cognition	Factor 3
WRAT-4: Math	[106]	Math Computation	1	General Ability	General Ability	*General Ability*	
WRAT-4: Reading	[106]	Word Reading	1	General Ability	General Ability	*General Ability*	
Vocabulary	[15]	Vocabulary	1,2	General Ability	Verbal Learning	*General Ability*	
WAIS-4: Digit Symbol	[24]	Complex Attention	1	Motor Speed	Motor Speed	*Motor Speed*	
Grooved Pegboard	[107]	Dexterity	1	Motor Speed	Motor Speed	*Motor Speed*	
Walk-a-Line	[33]	Ataxia	1	Balance	Balance	*Balance*	

Note. Factors that are consistent across models are shaded. Italics indicate factors that were supplements to the original model. Suite 1 is the full.

NCANDA test battery. Suite 2 is the Penn CNB. The Gur model is limited to only suite 2 assessments.

The present analysis aimed to replicate the previously reported factor structure of the Penn CNB [20] and extend these findings by testing model invariance. A second novel aim was to identify the optimal factor structure for the NCANDA neuropsychological battery when comparing candidate models, which are the 8-factor model from Sullivan et al. [15], the basic conceptual model (Gur Model) [20], the empirically derived factor analysis model (Empirical Model) [20], and a new model. The new model is one that prioritizes the neuropsychological functions reported to be the most vulnerable to alcohol exposure in human adolescents. To that end, a Vulnerabilities Model was developed and investigated. This model includes factors designed to tap visuospatial processing, attention, memory, and verbal learning, which are believed to be most sensitive to alcohol exposure in adolescence based on a review of existing published data [3]. Establishing a measurement model for these vulnerabilities could enhance the estimation of associations between target neuropsychological constructs and alcohol consumption. Accordingly, our overarching aim was to identify which factor structures are applicable to contemporary measurement batteries in youth samples in the context of substance use research.

## Materials and methods

### Sample

Data originate from the NCANDA project. NCANDA is a longitudinal study of community youth that employs a modified accelerated longitudinal design [12, 21, 22]. The main analyses presented in the current study are based on the measurements obtained at the baseline visit, which limits the influence of differential practice effects in this report. Recruitment and enrollment (n_participants_ = 831, n_families_ = 670) resulted in samples demographically representative of each metropolitan catchment region (n_regions_ = 5), with enrichment of participants at risk for lifetime substance use disorder; 51% of the participants reported at least one of the following: family history of alcohol use disorder, externalizing or internalizing symptoms, or consumption of alcohol before age 15. Exclusionary criteria included magnetic resonance imaging contraindications (e.g., permanent metal in the head such as dental braces), neurotropic medications, serious medical problems, major mental health disorder, including autism spectrum disorder (ASD), uncorrectable vision or hearing impairment, lack of English fluency, and substance use disorder. As the methodological details of the study design were previously published [12] only the core features of the protocol are presented here. Extensive quantitative descriptions of the neuropsychological scores can be found in Sullivan et al. [15]. At project baseline, participants were 12–21 years old, with oversampling of younger ages and those with no more than limited substance use experience. Participants’ neuropsychological functioning was assessed annually. The protocol was reviewed and approved by the human research protection program at each participating university and by the University of California San Diego Human Research Protections Program for the overall administration of the study. Both written informed parental consent and written informed child assent were obtained for juveniles and written informed consent obtained for participants who were at least 18 years old.

### Measures

Neuropsychological functioning was assessed with the Penn CNB [23], plus several traditional tests including the Word Reading and Arithmetic sub-tests of the Wide Range Achievement Test-4 (WRAT4) [24], Grooved Pegboard Test [25], and Fregly-Graybiel Walk-a-Line (FGWL) postural stability test [26, 27]. The pegboard tests manual dexterity with a timed score for the completion of a peg insertion task with each hand. The FGWL assesses ataxia. Penn CNB assesses a range of neuropsychological domains and provided 12 separate and composite test scores based on performance accuracy and speed, spanning various domains of functioning (Table 1). The Penn CNB was slightly modified from the standard Penn CNB distribution to optimize the sensitivity of the battery to detect the effects of alcohol exposure. This was done as recognition for NCANDA’s focus on the effects of alcohol exposure. Memory is a cognitive function thought to be sensitive to alcohol exposure in adolescence [3], and thus, greater discrimination among memory functions was sought. The modifications to the standard Penn CNB consisted of two test substitutions. To better distinguish immediate-recall and delayed-recall, delayed versions of Visual Object Learning and Penn Face Memory tests were substituted for the Penn Line Orientation Task and the Age Differentiation Task found in the standard Penn CNB. However, the immediate and delayed versions of the memory tests were found to have strongly correlated errors in this sample and were ultimately combined (meaned). Accuracy scores, which are available for the Penn CNB and the traditional tests, were used in this study.

Demographics, including sex and age, parental socio-economic status (income, occupation and educational attainment), were obtained during the baseline interview via self-report as described in Brown et al. [12]. Familial relationships among participants were also identified through proband and parental self-report.

### Statistical analysis

#### Overview

The analysis goal was to identify leading factor models. The statistical analyses stepped through the following phases. Phase 1 consisted of estimation of confirmatory factor analyses (CFA) [15, 20]. Phase 2 consisted of evaluation of the fit of individual models. Model comparison followed in Phase 3. Phase 4 included invariance testing of the superior model(s). These steps were run for the models of the full NCANDA battery and then again for the Penn-CNB-only subset of tests.

#### Phase 1: Confirmatory factor analysis

Using confirmatory factor analysis (CFA), models were fit to the baseline NCANDA data. The models were fit to the data in the following sequence. Configural CFA models were fit using maximum likelihood. Targeted models that failed to converge were reestimated with starting values from pooled models, with Newton-Raphson iterations, and run using Stata’s *difficult* algorithm. The reported loadings are based on standardized solutions of the CFA models. Model configurations are presented in Table 1. Neuropsychological scores were treated as reflexive indicators [28]. To account for the non-independence of participants within families, models were initially fit (using Stata’s *gsem*) with participants nested within families [29, 30]. This structure resulted in the failure of most models to converge, even with substantial mitigation. In response, the modelling approach was modified. Models were run in Stata 15.1 using the *sem* functions [31]. The model comparison results were based on bootstrap estimates, where each bootstrap sample consisted of only one participant from each family. Means of up to 1,000 bootstrap samples are reported. The final models used in each bootstrap sample estimation were allowed to run out to 100 iterations. If convergence was not achieved, the statistics were treated as missing. In models that included single indicators on latent constructs, the variances of the indicator(s) were constrained to a constant equal to one minus the reliability [32]. Reliability estimates were extracted from prior analyses [23, 33]. Latent variables with single indicators do not provide the measurement benefits resulting from the use of measurement models but can provide utility to the overall substantive investigation [34]. *A priori* loadings were available to be used in metric models for the Empirical Model [20]. Because the version of the Penn CNB used in the NCANDA study included two unconventional tests, these were left free in all metric models. All other parameters, including loading for traditional neuropsychological tests, were left unconstrained. All first order models were correlated traits models.

At baseline, >99% (n_Penn CNB_ = 828) of participants completed the full Penn CNB assessment battery, and 97% (n_NCANDA Battery_ = 806) completed all neuropsychological assessments. Missing data was deleted listwise (i.e. complete case analysis was employed), because the most common missing value pattern was missing either the entire computerized neuropsychological battery or the battery of traditional assessments (e.g.,75% of cases with any missing Penn CNB tests were missing all the Penn CNB tests). This pattern limits the benefit of missing value analysis, where there are no auxiliary variables available that can provide proxy information on the missing observations. Eleven participants were missing both the ataxia and math ability assessments. The employed analyses assume missing values are missing completely at random [35]. An alternative approach that used full-information maximum likelihood (FIML), which assumes data are missing at random [35] and utilizes all cases, was also employed in a separate series of analyses.

Data were assessed for normality before model estimation. Univariate distributions were evaluated through visualization with histograms and Q-Q plots, and estimation of higher order moments. Multivariate distributions were assessed via scatterplot matrices, estimation of Mahalanobis distances, and evaluation with the BACON algorithm, set to detected outliers beyond the 15^th^ percentile of the χ^2^ distribution [36, 37]. As no overt two-way curvilinear associations or grossly non-normal distributions were identified, data were left untransformed.

#### Phase 2: Model fit evaluation

Evaluation of model fit was conducted by gauging fit indices against conventions outlined by Hu and Bentler [38]. Bright-line application of conventional cut-points is avoided in recognition of the graded nature of model adequacy [39]. Further, the focus was made on comparative fits among models rather than the fit of individual models [40]. We used likelihood ratio tests to evaluate the level of significance between the target model and the saturated and base models, under a neoFisherian evaluation framework [41, 42]. Incremental fit was assessed with the comparative fit index (CFI). Absolute fit was assessed with root mean square error approximation (RMSEA) and the standardized root mean square residual (SRMR). Values for these were viewed in light of conventional cut-offs of CFI > 0.95, RMSEA < 0.05, and SRMR ≤ 0.08 for identifying well-fitting models [38, 43]. Models were deemed clearly unacceptable if CFI < 0.80, RMSEA > 0.10, and SRMR > 0.10 [38]. Although there are additional fit indices that can be used in model evaluation, we present a limited preselected set of indices with adequate statistical properties, coverage, and interpretability.

#### Phase 3: Model comparison

Similar to the approach taken by Fournet et al. [44], the analytical goal was anchored in model comparison rather than model development. Two nested sets of indicators were evaluated. The broadest group used all of the available neuropsychological tests for the NCANDA sample, this is referred to as the NCANDA suite (16 indicators). The conceptually motivated models were applied to this set as configural models (Table 1). Because the Gur Model did not originally incorporate all of the NCANDA assessments, it was extended by adopting the conceptual structure outlined by Sullivan et al. [15] for the additional assessments that are included in the full NCANDA suite. The extended model (Gur+) is denoted by a plus symbol. Because an aim of this paper, and the focus of prior empirical research, has been on the factor structure of the Penn CNB tests, a suite of models limited to the Penn CNB are also evaluated (10 indicators; see Table 1). All of the conceptual models are applied to both assessment suites. An empirically developed model was also available to provide a benchmark for comparisons. The empirically motivated 3-factor model (Empirical Model) was based on an exploratory factor analysis model of Penn CNB accuracy scores reported by Moore et al. [20]. Because loadings were available from the earlier report they were used to construct a metric version (constrained loadings only) of the Empirical Model [20]. There was no empirical basis to extend the Empirical Model to cover the full NCANDA assessment suite, so this model was estimated only in the Penn CNB suite.

The Empirical Model was included as a supplement to the conceptually grounded set of models. The decision to supplement the set of models was made after an initial investigation of the preplanned models. In accordance with recommended reporting practices, all of the post-hoc analyses were identified and interpreted as exploratory research steps [45, 46].

Model comparisons were primarily based on a set of penalized-likelihood selection criteria (AIC, BIC) [47], and supplemented by CFI, RMSEA, and SRMR [48–51]. Where discrepancies between AIC and BIC model selection occurred, BIC selection prevailed because it is more consistent with our objective of approximating the correct model and giving deference to parsimony, rather than optimizing a predictive model [50, 52, 53].

#### Phase 4: Invariance testing

Once optimal models were identified from among the candidate CFA models, configural invariance was evaluated. For this purpose, three series of multi-group CFA models with metric and scalar parameter constraints were fit for age groups (< 16.5 years old, ≥ 16.5 years old), self-identified sex, and measurement waves (baseline through year 4). Although configural model fit adequacy was considered necessary, it was not treated as sufficient [54]. Having the same pattern of salient factor loadings across groups was considered supportive of a configural invariance finding [55]. After configural invariance was ascertained, multiple-group CFAs were estimated. Evaluation of metric invariance began by estimating a model where all parameters were freely estimated. This was defined as the configural model, which was then compared with a model that included equality constraints on the loadings across groups (the metric model). A scalar model with constrained loadings and intercepts was next estimated. Intercepts for one of the indicators of each factor was constrained to zero to address identifiability in the group-CFA models.

Likelihood ratio tests comparing these nested models were used to gauge the significance of the added restrictions in each successive model [56]. However, this mode of evaluation is confounded by sample size [57]; it does not separately identify features or gauge the magnitude of misconfiguration. Thus, differences in the parameter estimates were also inspected to evaluate their contributions to the differences among groups. Further, an additional set of model fit statistics were inspected to gauge the change in model fit as a consequence of each successive set of constraints. Differences in CFI and RMSEA were evaluated in light of recommendations that ΔCFI and ΔRMSEA should not exceed -0.010 and 0.015, respectively [58–60] (cf. [61]). Deference was given to ΔCFI, as recommended by Sellbom and Tellegen [48]. Where measurement invariance was not achieved, model modification indices (MI) were used to assist in the identifications of parameters that may be contributing to worsening model fit [62]. Joint tests of modification indices (MI) were applied in each model, first with *Wald* tests of all free parameters, evaluating if they significantly vary across groups, then with *score* tests of parameters constrained to equality across the groups (for a description of these classes of hypothesis tests see [63]). Parameters in the joint tests were limited to the loadings in the configural and metric models and included intercepts in the scalar model. Partial invariance was investigated by refitting the model with parameters identified by the MI being freely estimated, without constraint. Interpretation of invariance violations among groups relied on inspection of loading patterns in each subset. Invariance evaluation was conducted to assess generalizability and to screen for mitigable mechanisms that are affecting model fit statistics; model fit indices are sensitive to noninvariance [58].

Invariance evaluation was supplemented with the use of alignment optimization [64]. Although the conditions under which alignment optimization proves to be the most appropriate analytic tool for application are still unclear, there are some conditions where early work identifies this method as valuable, for example when there are few noninvariant parameters in a measurement model [65, 66].

## Results

### Sample characteristics

At baseline, ages ranged from 12.1 to 22.0. The median was 15.9 years with the 24^th^ and 75^th^ percentile for age at 14.1 and 18.0. Boys accounted for 49% of the sample. Most participants represented the 670 families in the NCANDA sample as singletons (n_singletons_ = 531) with 17 families contributing more than two children to the sample (n_two siblings_ = 244, n_> 2 siblings_ = 56). Of all families, 47% had at least one parent with a post-baccalaureate degree. Most of the remaining families had a parent with an undergraduate degree (43% of families). The highest degree was a high school diploma or equivalent for 8% of families; 1% of families had parents without a diploma. At baseline, the mean number of days participants had used alcohol and cannabis during their lifetime was 9.2 (SD = 35.4) and 8.6 (SD = 85.3), respectively. See Brown and colleagues [12] for a detailed description of the sample.

### Model comparisons

Application of the conceptually-motivated CFA models under investigation in this study resulted in the identification of multiple models that had a constellation of moderately strong fit statistics when applied to the Penn CNB suite of neuropsychological tests. However, when applied to the full NCANDA suite, the fits were only marginally sufficient. Application of BIC as the model selection criterion, in this broader suite, identified the Gur+ Model as the superior model (Table 2). The RMSEA point estimate for the Gur+ Model was at 0.06 with an associated probability of the population value being under 0.05 that was low (P_close_ > 0.05). The baseline comparison statistic for this model was moderate; CFIs were 0.92. The residual magnitude statistic evidenced adequate fit; the SRMR was 0.04. The competing models were similar on this fit statistic (Table 2). In addition, the 8-Factor Model’s BIC was of similar magnitude at 0.8 points higher, which is an equivocal difference based on a 5 point rule for comparison [67]. Further, the 8-Factor Model had a substantially superior AIC (21.6 points lower).

**Table 2:** Confirmatory factor analysis model fit comparisons.

Test Suite	Full NCANDA			Penn CNB Tests Only			
Statistic\Models	8-Factor	Vulnerabilities	Gur+	8-Factor	Vulnerabilities	Gur	Empirical
**Model Number**	1	2	3	4	5	6	7
**N**	806	806	806	827	827	827	827
**Likelihood ratio tests**							
LR χ^2^ (saturated)	245.87	270.47	268.44	69.65	91.16	81.88	81.97
df (saturated)	79	77	84	27	25	29	30
P-value (saturated)	0.00	0.00	0.00	0.00	0.00	0.00	0.00
LR χ^2^ (baseline)	2372.10	2372.19	2372.16	829.65	829.69	830.10	831.87
df (baseline)	120	120	120	45	45	45	45
P-value (baseline)	0.00	0.00	0.00	0.00	0.00	0.00	0.00
**Information criteria** (lower is better)							
AIC	27642.5	27671.9	27664.1	18220.0	18246.9	18231.0	18219.1
BIC	27969.4	28007.7	27968.6	18391.1	18426.9	18393.0	18376.6
**Population error** (lower is better)							
RMSEA	0.057	0.062	0.058	0.049	0.063	0.052	0.051
90% CI L.L.	0.049	0.054	0.050	0.035	0.049	0.039	0.038
90% CI U.L.	0.065	0.07	0.066	0.063	0.077	0.066	0.064
P(RMSEA<0.05)	0.082	0.01	0.054	0.537	0.073	0.376	0.435
**Baseline comparison** (higher is better)							
CFI	0.925	0.914	0.918	0.946	0.916	0.933	0.934
TLI	0.888	0.866	0.883	0.909	0.848	0.896	0.901
**Residual magnitude** (lower is better)							
SRMR	0.038	0.042	0.040	0.033	0.040	0.037	0.037
**n bootstrap samples converging** (N = 1000)	1000	995	1000	1000	1000	1000	995

Note. Model fit statistics were obtained from 1000 bootstrap samples containing one randomly selected participant per family. Penn CNB Suite models excluded all indicators that were obtained outside of Penn CNB. All models are configural models. The metric version of the Empirical Model failed to converge in any iterations. TLI is the Tucker-Lewis Index.

**Fig 1:**
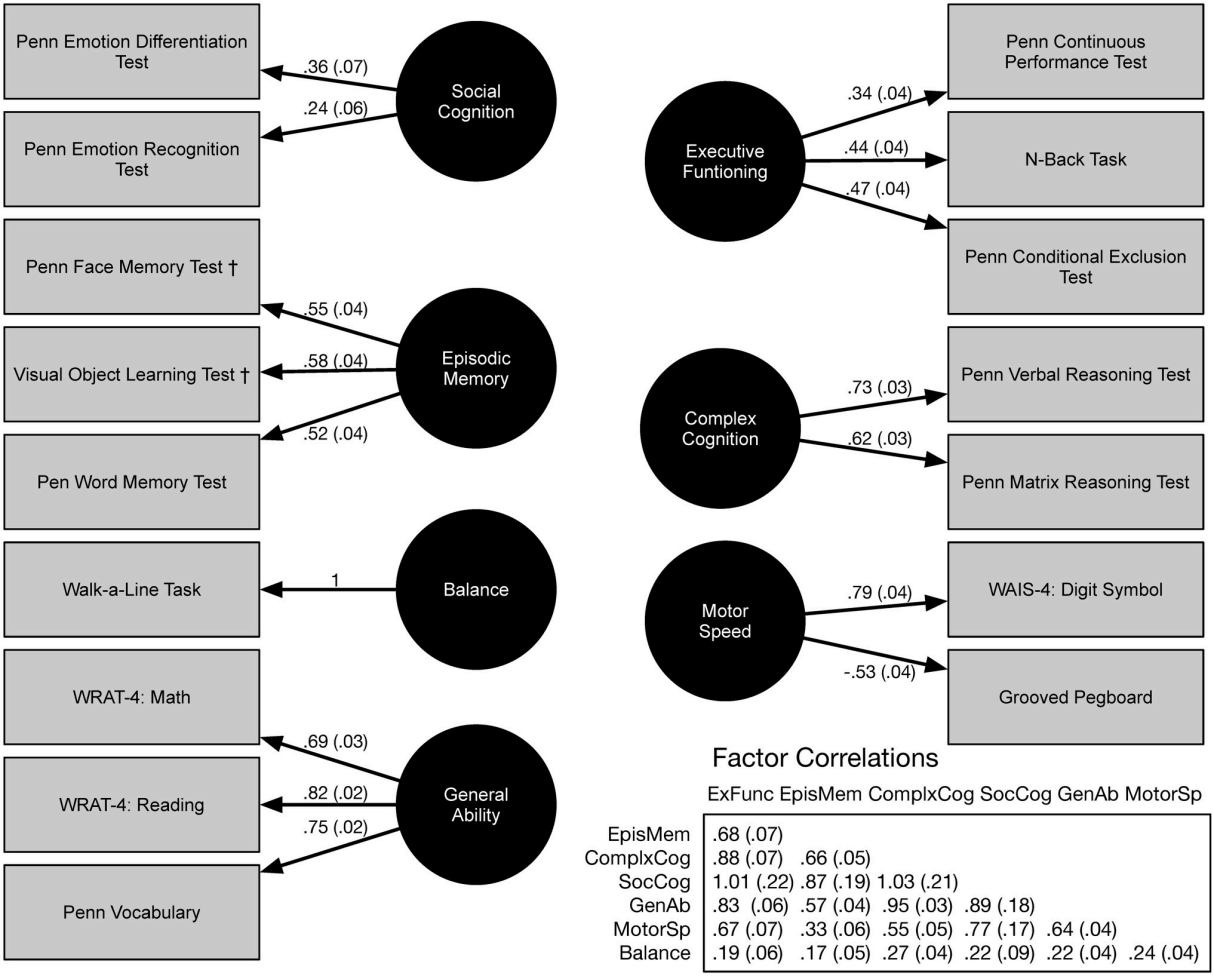
Confirmatory factor analysis estimates of the Gur+ Model for the NCANDA suite of tests.

The strongest loadings in the Gur+ Model were found with the General Ability, Motor Speed, and Complex Cognition latent factors (|λ|’s > 0.50, Fig 1). Episodic memory indictors were moderately loaded (λ’s of 0.52 to 0.58). Executive Function loadings were lower, especially for continuous performance (λ = 0.34). The only loading that was weaker was for the emotion recognition on Social Cognition (λ = 0.24). There was substantial correlation among the latent variables, with exception of postural stability (Balance) and the association between Motor Speed and Episodic Memory (Fig 1). The highest correlations were observed among Executive Functioning, Complex Cognition and Social Cognition (r’s > 0.88; Fig 1). There were two Heywood cases among the correlations with Social Cognition; the associated standard errors were much larger than the increment that the correlations exceeded one (Fig 1).

Model fit improved when the models were fit only to the Penn CNB tests (Table 2). Although the 8-Factor Model evidenced the lowest BIC among the conceptually motivated models (Models 1–6, Table 2), the Gur+ Model’s BIC was of similar magnitude (1.9 points higher). The 8-Factor Model demonstrated the best CFI, RMSEA, and SRMR for all the models estimated in this study (Table 2). At 0.95 and 0.05, respectively, the CFI and RMSEA values for the 8-Factor reached the borderline of good fit for factor models. Although the model fit statistics of the empirically derived factor model were slightly inferior to the 8-Factor Model, it had a superior BIC (14.5 points lower) and an equivalent AIC (0.9 points lower). Models estimated with FIML have similar fit statistics, although the Vulnerabilities and Empirical Models failed to converge (S1 Table).

**Fig 2:**
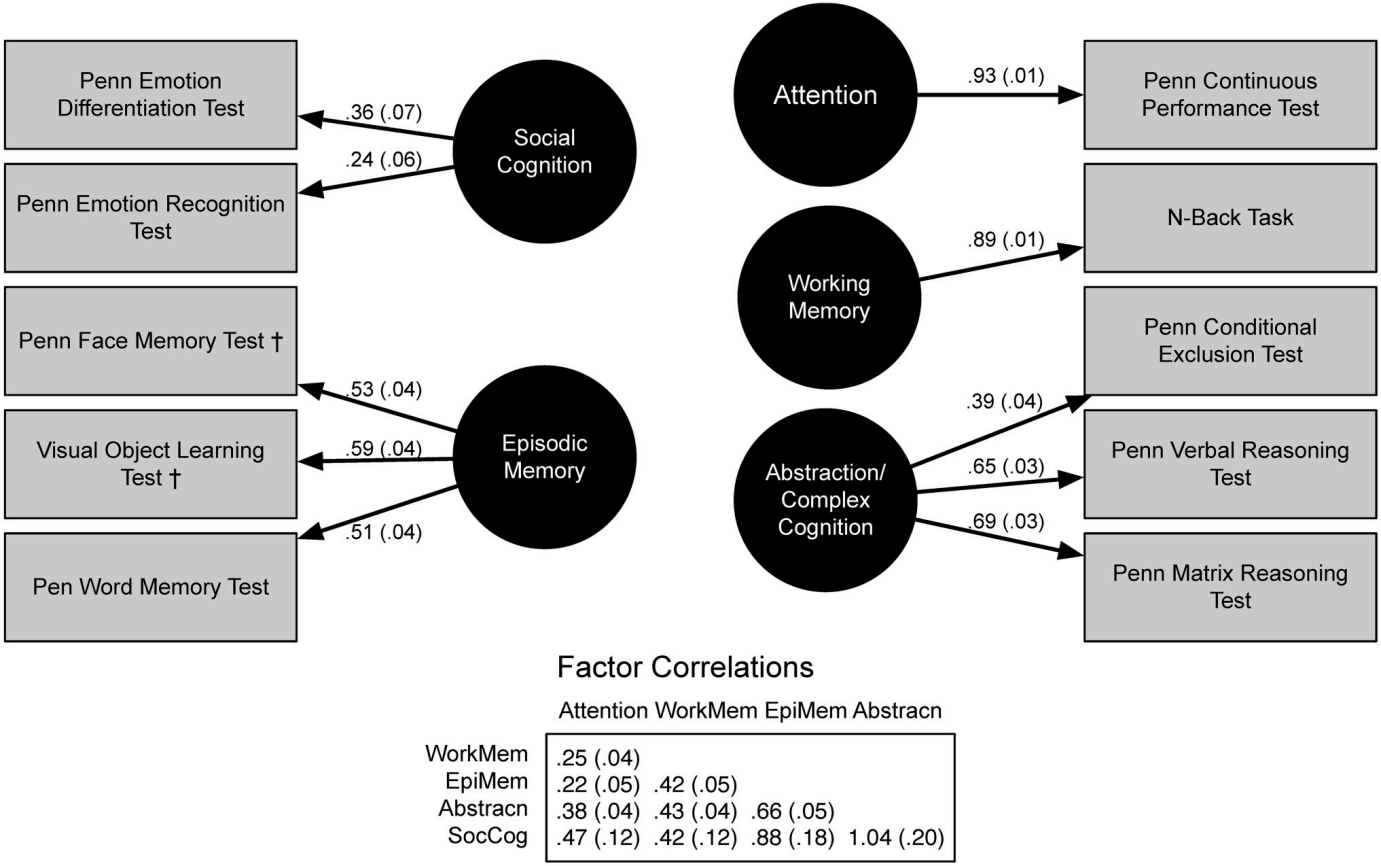
Confirmatory factor analysis estimates of the 8-Factor model for the Penn CNB suite of tests.

The 8-Factor Model had similar loading strengths as that observed in the Gur+ Model, for freely estimated loadings (Fig 2). The single indicator loadings were constrained by the estimated errors. The loading for the continuous exclusion test remained moderate in the 8-Factor model with a λ of 0.39. Correlations among the latent variables in this model were generally lower than observed in the Gur+ Model, with the notable exception of the correlations with Social Cognition (Fig 2). The correlation for Social Cognition and Abstraction was structured similarly to the Heywood cases observed in the Gur+ model (Fig 2).

In summary, when the CFA models were applied to the full NCANDA neuropsychological battery the Gur+ Model was selected based on the objective selection criteria for the full suite; however, it failed to demonstrate strong model fit, as did its competitors. Model fit for all the models applied to only the Penn CNB suite of tests improved, however the constellation of fit indices indicated they were at best at the borderline of being well fit. Overall, the best model fit indices were observed for the 8-Factor Model when it was applied only to the conventional Penn CNB tests. It also had favorable information criteria statistics. Only the Empirical Model had a better BIC. The Empirical Model’s fit statistics were equivalent to that observed for the 8-Factor Model.

### Invariance

Inspection of the salient loading patterns failed to identify substantial divergences in sex-, age-, or time point-specific models. Having evaluated configural invariance, metric and strong invariance by age, sex, and time point were evaluated in multiple-group CFA models. In the multi-group models evaluating age and time point, adding invariance constraints (metric and scalar) resulted in worsening of the models, based on likelihood ratio tests of nested models (*p*’s ≤ .05). Decrements in all the RMSEA values for the metric and scalar models were small (ΔRMSEA < 0.01), however the values all exceeded 0.05 (Table 3). This was most pronounced for the age models, which had point estimates near 0.10 (Table 3). In other words, RMSEA changes did not indicate notable worsening model fit with the invariance constraints, however the multi-group model fit was at best borderline based on this statistic. CFI patterns for age and time point were similar; for these groupings the metric model did not demonstrate a substantial worsening of the CFIs (ΔCFI ≤ 0.007). However, the scalar models did results in notable CFI decrements (ΔCFI ≥ 0.02). In other words, constraining intercepts to equality in the year and age group models resulted in worsening model fit as measured by CFI. In a partial scalar invariance model (Model A) the four most variant intercepts were freed. These four were for continuous performance, vocabulary, emotion recognition, and grooved pegboard tests. Intercepts for continuous performance were highest at baseline (ν = 0.26) and remained steady at the follow-ups (ν ≈ 0.16). Vocabulary and grooved pegboard both decline over time. The intercept for grooved pegboard consistently rose from 0.40 to -0.20 by year three. Alignment optimization failed to eliminate this noninvariance of intercepts (S1 File). The joint parameter tests also identified noninvariance by year and age (Table 3). Freeing the parameters increased the BIC and negligibly improved the CFI (Table 3). As with Model A, the partial scalar invariance model for age (Model B) insufficiently improved CFI to meet invariance criteria (Table 3). The partial invariance model for age also freed the most pronounced variant intercepts, which were for vocabulary, WRAT4: reading, and the n-back task. The intercepts for vocabulary and reading were lower by 0.39 and 0.33 for participants 17 or older as compared to younger participants, respectively. N-back scores were higher for younger participants by 0.53. Alignment optimization failed to substantially dampen these noninvariant intercepts (S1 File).

**Table 3:** Invariance tests for the Gur+ Model in the NCANDA suite.

Model	χ^2^ (df)	P-value	RMSEA (90%CI)	BIC	CFI	SRMR	ΔBIC	ΔCFI	ΔSRMR	Wald Tests (df)	Wald P-value	Score Tests (df)	Score P-value
Sex (girls, boys)													
Configural	457.39 (175)	0.000	0.070 (0.063, 0.078)	28294.3	0.876	0.055				9.9 (9)	0.372		
Metric	471.67 (184)	0.000	0.069 (0.062, 0.077)	28247.4	0.873	0.057	46.9	0.003	-0.002			13.6 (9)	0.157
Scalar	495.73 (193)	0.000	0.070 (0.062, 0.077)	28201.1	0.867	0.063	46.3	0.006	-0.006			7.57 (9)	0.588
Year (baseline, year 1, year 2, year 3)													
Configural	1142.29 (343)	0.000	0.065 (0.06, 0.069)	92792.1	0.906	0.049				51.01 (27)	0.008		
Metric	1197.16 (370)	0.000	0.063 (0.059, 0.067)	92635.8	0.902	0.054	156.3	0.004	-0.005			50.71 (27)	0.008
Scalar	1470.72 (397)	0.000	0.070 (0.066, 0.073)	92676.7	0.873	0.062	-40.9	0.029	-0.008			65.2 (27)	0.000
Partial Scalar A	1691.96 (456)	0.000	0.070 (0.066, 0.073)	95486.7	0.900	0.079	-2851	0.002	-0.025			82.45 (30)	0.000
Age (<16.5, > = 16.5)													
Configural	638.43 (175)	0.000	0.09 (0.083, 0.098)	28161.0	0.760	0.131				20.68 (9)	0.024		
Metric	662.16 (184)	0.000	0.090 (0.082, 0.097)	28130.3	0.753	0.135	30.7	0.007	-0.004			21.11 (9)	0.021
Scalar	739.99 (193)	0.000	0.093 (0.086, 0.101)	28137.0	0.717	0.162	-6.7	0.036	-0.027			41.07 (9)	0.000
Partial Scalar B	667.8 (190)	0.000	0.088 (0.081, 0.095)	28077.6	0.753	0.143	52.7	0.00	-0.008			20.13 (9)	0.028

Note. Partial Scalar Model A = scalar + Continuous Performance, Emotion Recognition, Word Memory, and Grooved Pegboard intercepts were freed, Partial Scalar Model B = scalar + N-Back, WRAT4: Reading, and Vocabulary intercepts were freed for baseline.

Although the model fit differences for the metric invariance models indicated adequate fit, the strongest score test for metric invariance was notable. The score test for the time point metric model was moderately significant (χ_(27)_ = 50.1, *p* = 0.011). Inspection of MIs and loadings both identified the most substantial loading variance was for working memory and attention indicators. In summary, pattern (metric) invariance was adequate for the Gur+ Model. Strong (scalar) invariance was not established for the Gur+ Model, but this noninvariance could partially mitigated through the freeing of limited sets of intercept parameters; however, in the case of the model for time point the SRMR and BIC worsened (Table 3).

The invariance patterns were similar for the 8-Factor model applied to the Penn CNB data (Table 4). An additional scalar noninvariance pattern was indicated by a marginal ΔBIC between the sex-grouped metric and scalar models (Table 4). However, none of the score tests indicated that the invariance patterns for sex and age could be distinguished from sampling error (*p*’s > 0.37). The freeing of the intercepts for the measures that demonstrated the greatest noninvariance in the partial invariance models rectified the ΔBIC’s in the scalar models grouped by time and age (Table 4). This relaxation allowed the intercepts for continuous exclusion to gradually rise 0.36 from baseline through to the 3^rd^ follow-up. Freeing of the intercepts for the continuous exclusion test in addition to facial memory and emotion recognition limited the remaining scalar noninvariance to acceptable levels (Table 4). Alignment optimization models failed to converge for the 8-factor model.

**Table 4:** Invariance tests for the 8-Factor Model applied to the Penn CNB test suite.

Model	χ^2^ (df)	P-value	RMSEA (90%CI)	BIC	CFI	SRMR	ΔBIC	ΔCFI	ΔSRMR	Wald Tests (df)	Wald P-value	Score Tests (df)	Score P-value
Sex (boys, girls)													
Configural	150.26 (59)	0.000	0.068 (0.055, 0.082)	18563.1	0.885	0.042				5.11 (5)	0.415		
Metric	157.31 (64)	0.000	0.066 (0.053, 0.079)	18534.0	0.883	0.045	29.1	0.002	-0.003			6.06 (5)	0.327
Scalar	174.6 (69)	0.000	0.068 (0.055, 0.08)	18526.8	0.867	0.046	7.2	0.016	-0.001			3.95 (5)	0.566
Partial Scalar C	173.31 (68)	0.000	0.068 (0.056, 0.081)	18530.1	0.867	0.046	3.9	0.016	-0.001			4.83 (5)	0.454
Year (baseline, year 1, year 2, year 3)													
Configural	295.99 (113)	0.000	0.053 (0.046, 0.061)	61218.3	0.945	0.033				24.84 (15)	0.079		
Metric	319.84 (128)	0.000	0.051 (0.044, 0.058)	61124.8	0.942	0.04	93.5	0.003	-0.007			23.47 (15)	0.105
Scalar	640.88 (143)	0.000	0.078 (0.072, 0.084)	61331.2	0.850	0.053	-206.4	0.092	-0.013			33.15 (15)	0.01
Partial Scalar D	348.77 (134)	0.000	0.053 (0.046, 0.06)	61107.4	0.935	0.041	17.4	0.007	-0.001			23.25 (15)	0.11
Age (<16.5, > = 16.5)													
Configural	217.85 (59)	0.000	0.09 (0.077, 0.103)	18476.0	0.776	0.068				5.33 (5)	0.401		
Metric	222.85 (64)	0.000	0.086 (0.074, 0.099)	18444.0	0.775	0.071	32	0.001	-0.003			5.25 (5)	0.414
Scalar	239.71 (69)	0.000	0.086 (0.074, 0.098)	18424.0	0.759	0.072	20	0.016	-0.001			5.61 (5)	0.376
Partial Scalar E	227.9 (67)	0.000	0.085 (0.073, 0.097)	18436.5	0.773	0.074	7.5	0.002	-0.003			4.97 (5)	0.438

Note. Partial Scalar Model C = scalar + Matrix Reasoning intercepts were freed, Model D = scalar + Emotion Recognition, Facial Memory, and Continuous Exclusion intercepts were freed, Model E = scalar + Continuous Exclusion and Visual Object Learning intercepts were freed.

## Discussion

### Model comparisons

We found support for the Gur+ Model when applied to the neuropsychological accuracy scores from NCANDA. It was the superior model for the full battery, based on the penalized-likelihood criteria. However, this model’s fit should be considered modest because the fit statistics were at best at the margins of acceptable levels [32, 38, 43]. When the suite of test scores entered into the factor model was restricted to the subset of tests obtained from NCANDA’s Penn CNB tests, the model fit statistics slightly improved. This was expected, in part, because the Gur Model was conceptually developed specifically for the Penn CNB [20]. The results from this study provide some support for the use of factor model configurations consistent with the conceptually motivated Gur Model.

The 8-Factor Model’s fit was similar to the Gur+ Model. The difference among these two models is limited to their treatment of executive functioning (Table 1); these models had the same factor structure except the 8-Factor Model separated out attention and working memory constructs as factors rather than grouping them into an executive functioning factor. In addition, the conditional exclusion test is placed within the Complex Cognition factor. Differences in models’ statistics reflect the relative merit of these configural distinctions.

Whereas the primary model selection statistic (BIC) identified the Gur+ Model for the full suite of NCANDA tests, its superiority over the 8-Factor Model was equivocal based on the small BIC difference. However, the 8-Factor Model’s fit statistics were slightly superior when applied to the Penn CNB suite of tests. Based on the statistical evaluation conducted here, researchers could justifiably interchange these models in order to optimally align the latent factors with the substantive targets of their investigations. This is a reasonable posture when the alternative models are equally supported in the descriptive literature (i.e., *phenomenological* models), and there is not a well-formed accepted hypothetico-deductive theory to derive a focusing model [68, 69]. Where alternative models are available, selection should optimize the trade-offs between realism, precision, and generality for the particular scientific question under investigation [70, 71]; under the perspective that no factor model is correct, the selection of an optimal approximating model should be based on context specific scientific considerations [72].

The 8-Factor Model does have several advantages over the Gur Model for application in the NCANDA study. The first is that it separates out neuropsychological constructs (working memory and attention) that have each been identified as being vulnerable to alcohol’s biological effects during adolescence [3, 73]. Second, this configuration is more aligned with the factor structure empirically identified for Penn CNB efficiency scores [20, 74]. This is because the 8-Factor model places the continuous exclusion test with verbal reasoning and matrix reasoning, which tap the Complex Cognition factor of the Gur Model. Finally, in some contexts the lower correlations among the latent constructs in the 8-Factor Model will provide improved discriminant capacity [75]. Although the findings presented in this study indicate that either of these two models can be reasonably be justified for use as a measurement model based on its individual model fit and conceptual underpinnings, the 8-Factor Model may prove to be of greatest utility for addressing the aims of the NCANDA study.

An empirically derived factor model was also evaluated in the current study. Moore et al. [20] reported an exploratory factor analysis model based on accuracy scores that we evaluated in a CFA framework. This model collapses the highly correlated Executive Functioning and Complex Cognition factors found in the Gur Model. It also parses the episodic memory and social cognition items differently (Table 1, Empirical Model). Providing some corroboration for Moore et al.’s earlier finding, the Empirical Model was the best model for the NCANDA Penn CNB suite based on BIC and was equivalent to the 8-Factor Model based on AIC (Table 2). The metric version of this model never converged in any of the bootstrap iterations, so only the configural structure is supported by the current study’s findings. NCANDA’s Penn CNB was modified from the standard version, in that it included two supplemental delayed recall tests and a modified nonverbal reasoning test and dropped a social cognition test. These differences could partially explain the discrepancies between the original loadings and loadings estimated in the current study that resulted in the lack of convergence.

### Invariance

Moore et al. [20] and James et al. [74] provided the only other published factor analysis of the standard Penn CNB. The findings presented here are consistent with the earlier reports. Moore estimated a CFI of 0.95 for the Gur Model applied to efficiency scores. This was higher (+0.032) than what was observed in this study. Support for the Gur model’s application is further strengthened by a number of favorable invariance findings. Based on CFI differentials, the Gur+ Model did not evidence substantial violations of metric invariance assumptions [48]. However, there was evidence of at least mild intercept noninvariance for time point and age, which could not be entirely mitigated in the partial scalar models or through alignment optimization. The scalar noninvariance indicates that some of the neuropsychology scores varied between groups in a way that diverged from the patterns of variability in the mean of the latent variable [76].

Two of the neuropsychology tests contributing to scalar invariance violations, were also associated with a moderately significant joint (score) test indicating some metric invariance violations are present across time points. These were the continuous performance test and the short fractal n-back test, which were designed as assessments of attention and working memory, respectively. These patterns may be partially attributable to differential practice effects that disrupt the correlation structure of the measurement battery. A prior report based on the NCANDA sample found these tests to be most sensitive to practice effects [77]. Rather than using raw test scores, application of test specific scores that are adjusted for test-specific practice effects could alleviate some temporal noninvariance. Additionally, neurocognitive domains (e.g. executive functioning and memory) and their component processes develop at different rates during childhood and adolescence, which could result in age related noninvariance [78, 79]. It is possible that the moderate model fit indices resulted from the observed deviations from noninvariance [58]. However, differential development of neuropsychological functioning would not be expected to contribute to noninvariance where the structure *within* factors is stationary as people age. Gur et al. did identify developmental patterns that were specific to individual Penn CNB tests [80]. For example, they found substantial and distinct improvements between childhood to early adulthood on the continuous performance test. This could necessitate the use of developmentally normed scores for longitudinal use of measurement models applied to Penn CNB scores, if the effects are found to be substantial in particular research settings.

Sensitivity analyses could be used to assess the importance of the observed magnitudes of scalar invariance and the benefit of applying mitigations such as multiple group factor analysis alignment [64, 76]. More importantly, distinct divergence in the developmental trajectories of individual indicators for putative factors should call into question the meaning of latent constructs created by factor analysis and our conceptualization of their connections to changes in the brain. It is up for debate, but some alternative approaches, such as network models, appear, at least on the surface, better able to address these complexities [81].

### Limitations

One of the current study’s limitations is the absence of an evaluation of potential changes in neuropsychological architecture as a result of alcohol exposure that would be represented as metric noninvariance. This is an important consideration in the context of the NCANDA project’s focus on effects of alcohol exposure, where such noninvariance might not be considered a statistical nuisance but instead be of material interest. The study design favoured substance use naïve participants at baseline [12], with few alcohol exposed participants recruited and only a small number transitioning into heavy episodic binge drinking patterns over the period analysed in this report [82]. The current study is not yet powered to detect subtle noninvariance associated with drinking exposure, but should be of substantial interest to future NCANDA investigators as the sample matures.

An important consideration of the current work is the limited number of indicators for some latent factors. Although, using single indicators can create psychometric [51] and computational challenges [83], their use may be necessitated by the underlying neuropsychological architecture and its match with the available measurements. Indeed, the NCANDA neuropsychological battery was designed to efficiently survey broad domains of neuropsychological functioning. This left some domains covered by a limited number of tests. In this study, when factors with single indicators were included, we used independent estimates of the error variance to determine the factor models in this study [32]. Irrespective of the quality of these variance estimates, this mitigation does not address the potential for construct underrepresentation [84], which cannot be mitigated without a study design change.

### Alternative models

Over time substantial diversity has developed in the conceptualized organization of cognitive abilities [85–91]. An important division of conceptualizations is found between researchers looking to understand neurological mechanisms and those looking to describe interindividual patterns of variation [81]. Concepts of intelligence have dominated the latter, and the other has roots in neuropathology. The positive manifold of cognitive ability tests has resulted in the frequent identification of a single common factor explaining a large portion of the variance among tests of cognitive ability [86, 92, 93]. This study’s neuropsychological assessment extends beyond neurological functioning addressed within the typical domains of intelligence research because of the focus on identifying broad neurobiological effects of adolescent alcohol use. For example, NCANDA measures postural stability in addition to domains commonly included in research on general cognitive abilities and individual variability in intelligence. Further, NCANDA’s test battery was not designed to optimize the measurement of latent constructs with multiple indicators but instead reflects the aspiration to maximize the breadth of neurologic functioning potentially affected by alcohol consumption. From the standpoint of measuring a general factor of intelligence, the test battery is biased [94].

Leading explanations for the positive manifold in the assessment of cognitive ability were founded on phenomenological models such as the *g*-factor, the sampling model, and the mutualism model [92, 95–97] (see Cummins [69] for a discussion of phenomenological models), which are incorporated into the integrated network model of van der Mass et al. [81]. This model posits that neurocognitive architecture can best be structured as crystallized and fluid cognitive abilities, as introduced by Cattell [87]. The biologically relevant distinction of this classification is supported by emerging data, such as those indicating differential heritability of crystalized and fluid cognitive abilities [98]. This architecture may be more consistent with brain function and development than is present in the explicit conceptualizations behind the sets of evaluated CFAs. Although no CFA models applied in this study were directly motivated by the Integrated Network Model, the CFAs have features consistent with the Integrated Network Model. For example, the results presented in this report point to a factor (general ability) containing all the crystallized cognitive ability tests in the better fitting models (Table 1). This suggests a possible benefit of future exploration of models influenced by Cattell for application.

## Conclusions

The study provides several contributions. It provides an independent corroboration of the Gur model and supports the adequacy of the Empirical Model to capture the factor structure of a computerized neuropsychological test battery that is easy to deploy and administer over the internet. Further, this is the first publication that includes formal invariance testing of the Penn CNB. The study also advances the approach of evaluating confirmatory factor models in a comparative framework, rather than looking at individual models in isolation. The comparisons in this study provide evidence that the conceptually derived model targeting neuropsychological processes posited to be most vulnerable to alcohol’s effects in adolescents (Vulnerabilities Model) was the most inferior model evaluated. This finding demonstrates one of the risks of employing ad hoc model configurations, without validation and comparison to alternative structures supported by theory and a body of prior work. As noted above, the 8-Factor model was found to be sufficient and evidenced slight superiority in model fit to the alternative models when applied to the Penn CNB Suite of neuropsychological tests. Although, there were marginal Heywood cases observed in the leading models, which is possible if models are correctly specified and estimated via maximum likelihood [99, 100], a confidence interval for these cases broadly covered admissible ranges. Thus, use of either of the leading models should be conducted with the recognition that Social Cognition is highly correlated with Complex Cognition and re-specification of the model to accommodate this aspect of the relationship could potentially improve some characteristics of the models. The variance in Social Cognition in the NCANDA sample is likely to be dampened by the exclusion of persons with ASD. Application of these models in a sample that includes persons with ASD may improve the discrimination between Social Cognition and other latent variables.

In the absence of an integrated theory of brain and neurocognitive functioning that functions as a well-formed engrained hypothetico-deductive theory we will continue to work with factor models that are influenced by research inertia and indeterminant competition among alternatives models. Work by authors such Patt et al. [18] on the structure of neuropsychology and others who are investigating the connections between alterations in brain and neuropsychological functioning as a consequences of exposure to substance use, infectious disease, and trauma [4, 9, 101–105] provides the empirical foundations necessary to achieve such theory. Until a theory is fully developed, some exploratory efforts to aptly describe the variance patterns within neuropsychological batteries should continue, even where a confirmatory factor model has been previously developed and found to fit in independent samples. These efforts can operate in a Bayesian framework (e.g., [78] or in an exploratory framework where series of exploratory factor models are compared (e.g., [18, 44]. The recommendations to continuously interweave exploratory factor analysis and confirmatory factor analysis into model building (see [51] would be most appropriate when conducting phenomenological modelling [68, 69], including in research contexts like the NCANDA project where the statistical superiority of alternative models is equivocal.

## Supporting information

S1 Table Confirmatory factor analysis model fit comparisons in models fit with FIML. (PDF)

S1 File Alignment optimization of the Gur+ Model by sex, time, and age. Alignment optimization was run in Mplus version 8.3.
(PDF)
