# Supplementary material for: Comparison of factor analysis models applied to the NCANDA neuropsychological test battery
Source: PLoS One. 2022 Feb 10;17(2):e0263174. doi: 10.1371/journal.pone.0263174 (PMC8830737; doi:10.1371/journal.pone.0263174)
Supplement: S1 Table — (PDF) [file pone.0263174.s001.pdf]

Statistic	8-Factor	Gur+	8-Factor	Gur
Model Number	1	3	4	6
LR Chi-square (saturated)	255.903	277.88699	68.238998	80.481003
df (saturated)	79	84	27	29
P-value (saturated)	0	0	0	0
LR Chi-square (baseline)	2401.0271	2402.051	830.72498	829.43799
df (baseline)	120	120	45	45
P-value (baseline)	0	0	0	0
AIC	28341.734	28361.533	18250.879	18253.654
BIC	28670.766	28668.031	18421.98	18415.754
RMSEA	0.058	0.059	0.048	0.051
RMSEA 90% CI L.L.	0.05	0.051	0.034	0.038
RMSEA 90% CI U.L.	0.066	0.066	0.062	0.065
P(RMSEA<0.05)	0.062	0.038	0.572	0.407
CFI	0.922	0.915	0.948	0.934
TLI	0.882	0.879	0.913	0.898
