# Supplementary material for: Comparison of factor analysis models applied to the NCANDA neuropsychological test battery
Source: PLoS One. 2022 Feb 10;17(2):e0263174. doi: 10.1371/journal.pone.0263174 (PMC8830737; doi:10.1371/journal.pone.0263174)
Supplement: S1 File — Alignment optimization was run in Mplus version 8.3. (PDF) [file pone.0263174.s002.pdf]

Alignment Optimization: Invariance Evaluation of the Gur+ CFA model (Model 3)

Variables

```
cnp_sf2b2_crc : short Fractal 2-back: correct pos and neg (weighted)
cnp_cpw_iwrd_tot : Word Memory: CPW Total Correct
cnp_pvrt_pvrtcr : Verbal Reasoning: Total Correct (verbal analogy/'logical reasoning')
cnp_pmat24a_pmat24_a_cr : Matrix Reasoning: PMAT Total Correct (Special FORM A)
cnp_medf36_medf36_a : Emotion Differentiation: Total Correct
cnp_er40d_er40_cr : Emotion Recognition: ER40 Total Correct
np_wrat4_mc_raw : WRAT:math (mc)
np_wrat4_wr_raw : WRAT:Word (wr)
cnp_pvoc_pvoccr : Vocabulary: Total Correct
np_wais4_rawscore : Raw Score for WAIS (digit symbol)
np_gpeg_timec : Dexterity: Composite Time on Grooved Pedboard
np_atax_sumc : Ataxia: sum of 4 sum scores
cnp_spcptnl_scpt_tp : Cont. Perform: Total True Positives (letter & num.)
cnp_pcet_pcetcr : PECT Accuracy2 = (PCET_CAT+1)*PCETCR/(PCETCR+PCETER)
meansvt : Mean of Visual Object Learning recall and delayed recall
meanfc : Mean of Face rec and delayed rec
```

Note: Models were run in Mplus version 8.3.

GUR+ Model: Invariance by Age
Age (12: 12-16; 17: 17+)

```
APPROXIMATE MEASUREMENT INVARIANCE (NONINVARIANCE) FOR GROUPS
 Intercepts/Thresholds
   CNP_SF2B    12 17
   CNP_CPW_    12 17
   CNP_PVRT    (12) (17)
   CNP_PMAT    12 17
   CNP_MEDF    12 17
   CNP_ER40    12 17
   NP_WRAT4    (12) (17)
   NP_WRAT4    (12) (17)
   CNP_PVOC    12 17
   NP_WAIS4    (12) (17)
   NP_GPEG_    12 17
   CNP_SPCP    (12) (17)
   CNP_PCET    (12) (17)
   MEANSVT     12 17
   MEANFC      (12) (17)
 Loadings for EXFUNC
   CNP_SF2B    12 17
   CNP_SPCP    12 17
   CNP_PCET    12 17
 Loadings for EPISMEM
   CNP_CPW_    12 17
   MEANSVT     12 17
   MEANFC      12 17
 Loadings for CMPLXCOG
   CNP_PVRT    12 17
   CNP_PMAT    12 17
 Loadings for SOCCOG
   CNP_MEDF    12 17
   CNP_ER40    12 17
 Loadings for GENAB
   NP_WRAT4    12 17
   NP_WRAT4    12 17
   CNP_PVOC    (12) (17)
 Loadings for MOTORSP
   NP_WAIS4    12 17
```

```
   NP_GPEG_    12 17
FACTOR MEAN COMPARISON AT THE 5% SIGNIFICANCE LEVEL IN DESCENDING ORDER
 Results for Factor EXFUNC
           Latent    Group      Factor
 Ranking    Class    Value       Mean     Groups With Significantly Smaller Factor Mean
     1         1        12       0.000
     2         2        17      -0.021
 Results for Factor EPISMEM
           Latent    Group      Factor
 Ranking    Class    Value       Mean     Groups With Significantly Smaller Factor Mean
     1         1        12       0.000    17
     2         2        17      -0.177
 Results for Factor CMPLXCOG
           Latent    Group      Factor
 Ranking    Class    Value       Mean     Groups With Significantly Smaller Factor Mean
     1         2        17       0.122
     2         1        12       0.000
 Results for Factor SOCCOG
           Latent    Group      Factor
 Ranking    Class    Value       Mean     Groups With Significantly Smaller Factor Mean
     1         2        17       0.203
     2         1        12       0.000
 Results for Factor GENAB
           Latent    Group      Factor
 Ranking    Class    Value       Mean     Groups With Significantly Smaller Factor Mean
     1         2        17       0.355    12
     2         1        12       0.000
 Results for Factor MOTORSP
           Latent    Group      Factor
 Ranking    Class    Value       Mean     Groups With Significantly Smaller Factor Mean
     1         1        12       0.000
     2         2        17      -0.051
ALIGNMENT OUTPUT
INVARIANCE ANALYSIS
 Intercepts/Thresholds
 Intercept for CNP_SF2B
 Group     Group      Value      Value     Difference  SE       P-value
    17        12     -0.116      0.043     -0.159      0.088      0.070
 Approximate Measurement Invariance Holds For Groups:
 12 17
 Weighted Average Value Across Invariant Groups:      -0.029
 R-square/Explained variance/Invariance index:       0.150
 Invariant Group Values, Difference to Average and Significance
 Group      Value Difference         SE    P-value
    12      0.043      0.071      0.039      0.070
    17     -0.116     -0.088      0.048      0.070
 Intercept for CNP_CPW_
 Group     Group      Value      Value     Difference  SE       P-value
    17        12      0.227      0.239     -0.012      0.048      0.798
 Approximate Measurement Invariance Holds For Groups:
 12 17
 Weighted Average Value Across Invariant Groups:       0.234
 R-square/Explained variance/Invariance index:       0.997
 Invariant Group Values, Difference to Average and Significance
 Group      Value Difference         SE    P-value
    12      0.239      0.005      0.021      0.798
    17      0.227     -0.007      0.026      0.798
 Intercept for CNP_PVRT
 Group     Group      Value      Value     Difference  SE       P-value
 Approximate Invariance Was Not Found For This Parameter.
 Intercept for CNP_PMAT
 Group     Group      Value      Value     Difference  SE       P-value
    17        12     -0.077     -0.130      0.053      0.017      0.002
```

```
Approximate Measurement Invariance Holds For Groups:
12 17
Weighted Average Value Across Invariant Groups:      -0.106
R-square/Explained variance/Invariance index:       0.823
Invariant Group Values, Difference to Average and Significance
Group      Value Difference         SE    P-value
    12     -0.130     -0.024      0.008      0.002
    17     -0.077      0.029      0.009      0.002
Intercept for CNP_MEDF
Group     Group      Value      Value     Difference  SE       P-value
    17        12     -0.026     -0.003     -0.023      0.013      0.081
Approximate Measurement Invariance Holds For Groups:
12 17
Weighted Average Value Across Invariant Groups:      -0.013
R-square/Explained variance/Invariance index:       0.884
Invariant Group Values, Difference to Average and Significance
Group      Value Difference         SE    P-value
    12     -0.003      0.010      0.006      0.081
    17     -0.026     -0.013      0.007      0.081
Intercept for CNP_ER40
Group     Group      Value      Value     Difference  SE       P-value
    17        12     -0.235     -0.268      0.033      0.086      0.699
Approximate Measurement Invariance Holds For Groups:
12 17
Weighted Average Value Across Invariant Groups:      -0.253
R-square/Explained variance/Invariance index:       0.907
Invariant Group Values, Difference to Average and Significance
Group      Value Difference         SE    P-value
    12     -0.268     -0.015      0.039      0.699
    17     -0.235      0.018      0.048      0.699
Intercept for NP_WRAT4
Group     Group      Value      Value     Difference  SE       P-value
Approximate Invariance Was Not Found For This Parameter.
Intercept for NP_WRAT4
Group     Group      Value      Value     Difference  SE       P-value
    17        12     -0.225     -0.201     -0.024      0.005      0.000
Approximate Invariance Was Not Found For This Parameter.
Intercept for CNP_PVOC
Group     Group      Value      Value     Difference  SE       P-value
    17        12     -0.177     -0.266      0.089      0.072      0.213
Approximate Measurement Invariance Holds For Groups:
12 17
Weighted Average Value Across Invariant Groups:      -0.226
R-square/Explained variance/Invariance index:       0.885
Invariant Group Values, Difference to Average and Significance
Group      Value Difference         SE    P-value
    12     -0.266     -0.040      0.032      0.213
    17     -0.177      0.049      0.040      0.213
Intercept for NP_WAIS4
Group     Group      Value      Value     Difference  SE       P-value
Approximate Invariance Was Not Found For This Parameter.
Intercept for NP_GPEG_
Group     Group      Value      Value     Difference  SE       P-value
    17        12      0.179      0.289     -0.110      0.105      0.293
Approximate Measurement Invariance Holds For Groups:
12 17
Weighted Average Value Across Invariant Groups:       0.240
R-square/Explained variance/Invariance index:       0.428
Invariant Group Values, Difference to Average and Significance
Group      Value Difference         SE    P-value
    12      0.289      0.049      0.047      0.293
    17      0.179     -0.061      0.058      0.293
Intercept for CNP_SPCP
```

```
Group     Group      Value      Value     Difference  SE       P-value
    17        12      0.016     -0.357      0.373      0.089      0.000
Approximate Invariance Was Not Found For This Parameter.
Intercept for CNP_PCET
Group     Group      Value      Value     Difference  SE       P-value
Approximate Invariance Was Not Found For This Parameter.
Intercept for MEANSVT
Group     Group      Value      Value     Difference  SE       P-value
    17        12      0.089      0.111     -0.022      0.053      0.683
Approximate Measurement Invariance Holds For Groups:
12 17
Weighted Average Value Across Invariant Groups:       0.101
R-square/Explained variance/Invariance index:       0.937
Invariant Group Values, Difference to Average and Significance
Group      Value Difference         SE    P-value
    12      0.111      0.010      0.024      0.683
    17      0.089     -0.012      0.029      0.683
Intercept for MEANFC
Group     Group      Value      Value     Difference  SE       P-value
Approximate Invariance Was Not Found For This Parameter.
Loadings for CNP_SF2B
Group     Group      Value      Value     Difference  SE       P-value
    17        12      0.638      0.649     -0.011      0.019      0.567
Approximate Measurement Invariance Holds For Groups:
12 17
Weighted Average Value Across Invariant Groups:       0.644
R-square/Explained variance/Invariance index:       0.998
Invariant Group Values, Difference to Average and Significance
Group      Value Difference         SE    P-value
    12      0.649      0.005      0.009      0.567
    17      0.638     -0.006      0.011      0.567
Loadings for CNP_SPCP
Group     Group      Value      Value     Difference  SE       P-value
    17        12      0.402      0.631     -0.228      0.128      0.074
Approximate Measurement Invariance Holds For Groups:
12 17
Weighted Average Value Across Invariant Groups:       0.528
R-square/Explained variance/Invariance index:       0.733
Invariant Group Values, Difference to Average and Significance
Group      Value Difference         SE    P-value
    12      0.631      0.103      0.057      0.074
    17      0.402     -0.126      0.071      0.074
Loadings for CNP_PCET
Group     Group      Value      Value     Difference  SE       P-value
    17        12      0.594      0.531      0.063      0.143      0.659
Approximate Measurement Invariance Holds For Groups:
12 17
Weighted Average Value Across Invariant Groups:       0.559
R-square/Explained variance/Invariance index:       0.860
Invariant Group Values, Difference to Average and Significance
Group      Value Difference         SE    P-value
    12      0.531     -0.028      0.064      0.659
    17      0.594      0.035      0.079      0.659
Loadings for CNP_CPW_
Group     Group      Value      Value     Difference  SE       P-value
    17        12      0.535      0.604     -0.069      0.110      0.531
Approximate Measurement Invariance Holds For Groups:
12 17
Weighted Average Value Across Invariant Groups:       0.573
R-square/Explained variance/Invariance index:       0.883
Invariant Group Values, Difference to Average and Significance
Group      Value Difference         SE    P-value
    12      0.604      0.031      0.049      0.531
```

```
    17      0.535     -0.038      0.061      0.531
 Loadings for MEANSVT
 Group     Group      Value      Value     Difference  SE       P-value
    17        12      0.786      0.577      0.208      0.140      0.137
 Approximate Measurement Invariance Holds For Groups:
 12 17
 Weighted Average Value Across Invariant Groups:       0.671
 R-square/Explained variance/Invariance index:       0.000
 Invariant Group Values, Difference to Average and Significance
 Group      Value Difference         SE    P-value
    12      0.577     -0.093      0.063      0.137
    17      0.786      0.115      0.077      0.137
 Loadings for MEANFC
 Group     Group      Value      Value     Difference  SE       P-value
    17        12      0.697      0.692      0.005      0.010      0.597
 Approximate Measurement Invariance Holds For Groups:
 12 17
 Weighted Average Value Across Invariant Groups:       0.695
 R-square/Explained variance/Invariance index:       0.999
 Invariant Group Values, Difference to Average and Significance
 Group      Value Difference         SE    P-value
    12      0.692     -0.002      0.004      0.597
    17      0.697      0.003      0.005      0.597
Loadings for CNP_PVRT
 Group     Group      Value      Value     Difference  SE       P-value
    17        12      0.773      0.829     -0.055      0.055      0.316
 Approximate Measurement Invariance Holds For Groups:
 12 17
 Weighted Average Value Across Invariant Groups:       0.804
 R-square/Explained variance/Invariance index:       0.828
 Invariant Group Values, Difference to Average and Significance
 Group      Value Difference         SE    P-value
    12      0.829      0.025      0.025      0.316
    17      0.773     -0.030      0.030      0.316
 Loadings for CNP_PMAT
 Group     Group      Value      Value     Difference  SE       P-value
    17        12      0.655      0.612      0.043      0.042      0.308
 Approximate Measurement Invariance Holds For Groups:
 12 17
 Weighted Average Value Across Invariant Groups:       0.631
 R-square/Explained variance/Invariance index:       0.000
 Invariant Group Values, Difference to Average and Significance
 Group      Value Difference         SE    P-value
    12      0.612     -0.019      0.019      0.308
    17      0.655      0.024      0.023      0.308
Loadings for CNP_MEDF
 Group     Group      Value      Value     Difference  SE       P-value
    17        12      0.416      0.395      0.021      0.017      0.206
 Approximate Measurement Invariance Holds For Groups:
 12 17
 Weighted Average Value Across Invariant Groups:       0.404
 R-square/Explained variance/Invariance index:       0.681
 Invariant Group Values, Difference to Average and Significance
 Group      Value Difference         SE    P-value
    12      0.395     -0.009      0.008      0.206
    17      0.416      0.012      0.009      0.206
 Loadings for CNP_ER40
 Group     Group      Value      Value     Difference  SE       P-value
    17        12      0.179      0.299     -0.120      0.102      0.241
 Approximate Measurement Invariance Holds For Groups:
 12 17
 Weighted Average Value Across Invariant Groups:       0.245
 R-square/Explained variance/Invariance index:       0.000
```

```
 Invariant Group Values, Difference to Average and Significance
 Group      Value Difference         SE    P-value
     12      0.299      0.054      0.046      0.241
     17      0.179     -0.066      0.056      0.241
Loadings for NP_WRAT4
 Group     Group      Value      Value     Difference  SE       P-value
     17        12      0.769      0.770     -0.001      0.027      0.981
 Approximate Measurement Invariance Holds For Groups:
 12 17
 Weighted Average Value Across Invariant Groups:       0.769
 R-square/Explained variance/Invariance index:       1.000
 Invariant Group Values, Difference to Average and Significance
 Group      Value Difference         SE    P-value
     12      0.770      0.000      0.012      0.981
     17      0.769      0.000      0.015      0.981
 Loadings for NP_WRAT4
 Group     Group      Value      Value     Difference  SE       P-value
     17        12      0.868      0.924     -0.056      0.072      0.442
 Approximate Measurement Invariance Holds For Groups:
 12 17
 Weighted Average Value Across Invariant Groups:       0.899
 R-square/Explained variance/Invariance index:       0.938
 Invariant Group Values, Difference to Average and Significance
 Group      Value Difference         SE    P-value
     12      0.924      0.025      0.032      0.442
     17      0.868     -0.031      0.040      0.442
 Loadings for CNP_PVOC
 Group     Group      Value      Value     Difference  SE       P-value
     17        12      0.942      0.637      0.305      0.092      0.001
 Approximate Invariance Was Not Found For This Parameter.
Loadings for NP_WAIS4
 Group     Group      Value      Value     Difference  SE       P-value
     17        12     -1.074     -0.967     -0.106      0.061      0.082
 Approximate Measurement Invariance Holds For Groups:
 12 17
 Weighted Average Value Across Invariant Groups:      -1.015
 R-square/Explained variance/Invariance index:       0.943
 Invariant Group Values, Difference to Average and Significance
 Group      Value Difference         SE    P-value
     12     -0.967      0.048      0.027      0.082
     17     -1.074     -0.059      0.034      0.082
 Loadings for NP_GPEG_
 Group     Group      Value      Value     Difference  SE       P-value
     17        12      0.641      0.731     -0.090      0.301      0.766
 Approximate Measurement Invariance Holds For Groups:
 12 17
 Weighted Average Value Across Invariant Groups:       0.691
 R-square/Explained variance/Invariance index:       0.962
 Invariant Group Values, Difference to Average and Significance
 Group      Value Difference         SE    P-value
     12      0.731      0.040      0.135      0.766
     17      0.641     -0.049      0.166      0.766
 Average Invariance index:       0.720
```

GUR+ Model: Invariance by Time

Time (0: Baseline; 1: Year 1; 2: Year 2; 3: Year 3)

```
 APPROXIMATE MEASUREMENT INVARIANCE (NONINVARIANCE) FOR GROUPS

   Intercepts/Thresholds
     CNP_SF2B    0 1 2 3
     CNP_CPW_    0 1 (2) (3)
```

```
   CNP_PVRT    (0) (1) (2) (3)
   CNP_PMAT    0 1 2 3
   CNP_MEDF    0 1 2 3
   CNP_ER40    (0) 1 2 3
   NP_WRAT4    (0) (1) (2) (3)
   NP_WRAT4    0 1 2 3
   CNP_PVOC    0 1 2 3
   NP_WAIS4    (0) (1) (2) (3)
   NP_GPEG_    (0) (1) 2 3
   CNP_SPCP    0 1 (2) (3)
   CNP_PCET    (0) (1) (2) (3)
   MEANSVT     0 1 2 3
   MEANFC      (0) (1) (2) (3)

 Loadings for EXFUNC
   CNP_SF2B    0 1 2 3
   CNP_SPCP    (0) 1 2 3
   CNP_PCET    0 1 2 3

 Loadings for EPISMEM
   CNP_CPW_    0 1 2 3
   MEANSVT     0 1 2 3
   MEANFC      0 1 2 3

 Loadings for CMPLXCOG
   CNP_PVRT    0 1 2 3
   CNP_PMAT    0 1 2 3

 Loadings for SOCCOG
   CNP_MEDF    0 1 2 3
   CNP_ER40    0 1 2 3

 Loadings for GENAB
   NP_WRAT4    0 1 2 3
   NP_WRAT4    0 1 2 3
   CNP_PVOC    0 1 2 3

 Loadings for MOTORSP
   NP_WAIS4    (0) 1 2 3
   NP_GPEG_    (0) 1 2 3


FACTOR MEAN COMPARISON AT THE 5% SIGNIFICANCE LEVEL IN DESCENDING ORDER

 Results for Factor EXFUNC

          Latent    Group      Factor
 Ranking    Class    Value       Mean     Groups With Significantly Smaller Factor Mean
     1        2        1       0.137
     2        4        3       0.112
     3        3        2       0.092
     4        1        0       0.000

 Results for Factor EPISMEM

          Latent    Group      Factor
 Ranking    Class    Value       Mean     Groups With Significantly Smaller Factor Mean
     1        1        0       0.000    3
     2        2        1      -0.040    3
     3        3        2      -0.076
     4        4        3      -0.184

 Results for Factor CMPLXCOG
```

Ranking	Latent Class	Group Value	Factor Mean	Groups With Significantly Smaller Factor Mean
1	4	3	0.101	0
2	3	2	0.048	
3	2	1	0.029	
4	1	0	0.000	

Results for Factor SOCCOG

Ranking	Latent Class	Group Value	Factor Mean	Groups With Significantly Smaller Factor Mean
1	1	0	0.000	
2	3	2	-0.071	
3	2	1	-0.119	
4	4	3	-0.194	

Results for Factor GENAB

Ranking	Latent Class	Group Value	Factor Mean	Groups With Significantly Smaller Factor Mean
1	4	3	0.124	1 0
2	3	2	0.070	0
3	2	1	0.007	
4	1	0	0.000	

Results for Factor MOTORSP

Ranking	Latent Class	Group Value	Factor Mean	Groups With Significantly Smaller Factor Mean
1	4	3	0.021	1 0
2	3	2	0.011	1 0
3	2	1	0.003	
4	1	0	0.000	

ALIGNMENT OUTPUT

INVARIANCE ANALYSIS

Intercepts/Thresholds
Intercept for CNP_SF2B

Group	Group	Value	Value	Difference	SE	P-value
1	0	-0.004	-0.076	0.072	0.071	0.310
2	0	-0.063	-0.076	0.013	0.022	0.560
2	1	-0.063	-0.004	-0.059	0.060	0.328
3	0	-0.005	-0.076	0.071	0.073	0.329
3	1	-0.005	-0.004	-0.001	0.029	0.970
3	2	-0.005	-0.063	0.058	0.059	0.322

Approximate Measurement Invariance Holds For Groups:
0 1 2 3
Weighted Average Value Across Invariant Groups: -0.039
R-square/Explained variance/Invariance index: 0.659

Invariant Group Values, Difference to Average and Significance

Group	Value	Difference	SE	P-value
0	-0.076	-0.037	0.037	0.319
1	-0.004	0.035	0.036	0.334
2	-0.063	-0.024	0.025	0.341
3	-0.005	0.034	0.037	0.358

Intercept for CNP_CPW_

Group	Group	Value	Value	Difference	SE	P-value
1	0	0.047	0.164	-0.117	0.053	0.026

2	0	-0.083	0.164	-0.247	0.053	0.000
2	1	-0.083	0.047	-0.129	0.049	0.008
3	0	-0.105	0.164	-0.269	0.056	0.000
3	1	-0.105	0.047	-0.152	0.052	0.004
3	2	-0.105	-0.083	-0.023	0.043	0.595

Approximate Measurement Invariance Holds For Groups:
0 1
Weighted Average Value Across Invariant Groups: 0.108
R-square/Explained variance/Invariance index: 0.404

Invariant Group Values, Difference to Average and Significance

Group	Value	Difference	SE	P-value
0	0.164	0.056	0.025	0.026
1	0.047	-0.061	0.028	0.026

Intercept for CNP_PVRT

Group	Group	Value	Value	Difference	SE	P-value

Approximate Measurement Invariance Holds For Groups:

Weighted Average Value Across Invariant Groups: NaN
R-square/Explained variance/Invariance index: 0.000

Invariant Group Values, Difference to Average and Significance

Group	Value	Difference	SE	P-value

Intercept for CNP_PMAT

Group	Group	Value	Value	Difference	SE	P-value
1	0	-0.039	-0.055	0.016	0.022	0.476
2	0	-0.028	-0.055	0.027	0.022	0.215
2	1	-0.028	-0.039	0.011	0.022	0.601
3	0	-0.002	-0.055	0.053	0.021	0.013
3	1	-0.002	-0.039	0.038	0.022	0.082
3	2	-0.002	-0.028	0.026	0.022	0.236

Approximate Measurement Invariance Holds For Groups:
0 1 2 3
Weighted Average Value Across Invariant Groups: -0.033
R-square/Explained variance/Invariance index: 0.785

Invariant Group Values, Difference to Average and Significance

Group	Value	Difference	SE	P-value
0	-0.055	-0.022	0.013	0.082
1	-0.039	-0.006	0.013	0.626
2	-0.028	0.005	0.014	0.714
3	-0.002	0.031	0.014	0.024

Intercept for CNP_MEDF

Group	Group	Value	Value	Difference	SE	P-value
1	0	0.034	0.045	-0.011	0.007	0.106
2	0	0.024	0.045	-0.021	0.005	0.000
2	1	0.024	0.034	-0.011	0.005	0.046
3	0	0.001	0.045	-0.044	0.025	0.083
3	1	0.001	0.034	-0.033	0.021	0.111
3	2	0.001	0.024	-0.023	0.023	0.333

Approximate Measurement Invariance Holds For Groups:
0 1 2 3
Weighted Average Value Across Invariant Groups: 0.028
R-square/Explained variance/Invariance index: 0.888

Invariant Group Values, Difference to Average and Significance

Group	Value	Difference	SE	P-value
0	0.045	0.017	0.008	0.021
1	0.034	0.007	0.004	0.074
2	0.024	-0.004	0.006	0.509

3	0.001	-0.027	0.018	0.143

Intercept for CNP_ER40

Group	Group	Value	Value	Difference	SE	P-value
1	0	0.009	-0.233	0.242	0.064	0.000
2	0	0.169	-0.233	0.402	0.057	0.000
2	1	0.169	0.009	0.160	0.056	0.004
3	0	0.190	-0.233	0.423	0.059	0.000
3	1	0.190	0.009	0.181	0.056	0.001
3	2	0.190	0.169	0.021	0.039	0.587

Approximate Measurement Invariance Holds For Groups:
1 2 3
Weighted Average Value Across Invariant Groups: 0.117
R-square/Explained variance/Invariance index: 0.000

Invariant Group Values, Difference to Average and Significance

Group	Value	Difference	SE	P-value
1	0.009	-0.108	0.033	0.001
2	0.169	0.052	0.027	0.053
3	0.190	0.073	0.028	0.009

Intercept for NP_WRAT4

Group	Group	Value	Value	Difference	SE	P-value

Approximate Measurement Invariance Holds For Groups:

Weighted Average Value Across Invariant Groups: NaN
R-square/Explained variance/Invariance index: 0.000

Invariant Group Values, Difference to Average and Significance

Group	Value	Difference	SE	P-value

Intercept for NP_WRAT4

Group	Group	Value	Value	Difference	SE	P-value
1	0	-0.046	-0.069	0.024	0.030	0.430
2	0	0.011	-0.069	0.080	0.034	0.020
2	1	0.011	-0.046	0.056	0.032	0.078
3	0	0.059	-0.069	0.128	0.040	0.001
3	1	0.059	-0.046	0.104	0.038	0.006
3	2	0.059	0.011	0.048	0.035	0.170

Approximate Measurement Invariance Holds For Groups:
0 1 2 3
Weighted Average Value Across Invariant Groups: -0.016
R-square/Explained variance/Invariance index: 0.710

Invariant Group Values, Difference to Average and Significance

Group	Value	Difference	SE	P-value
0	-0.069	-0.053	0.020	0.009
1	-0.046	-0.029	0.019	0.123
2	0.011	0.027	0.020	0.182
3	0.059	0.075	0.026	0.004

Intercept for CNP_PVOC

Group	Group	Value	Value	Difference	SE	P-value
1	0	-0.034	-0.027	-0.007	0.021	0.740
2	0	-0.030	-0.027	-0.003	0.016	0.868
2	1	-0.030	-0.034	0.004	0.018	0.803
3	0	-0.029	-0.027	-0.002	0.012	0.857
3	1	-0.029	-0.034	0.005	0.014	0.723
3	2	-0.029	-0.030	0.000	0.016	0.977

Approximate Measurement Invariance Holds For Groups:
0 1 2 3
Weighted Average Value Across Invariant Groups: -0.030
R-square/Explained variance/Invariance index: 0.994

Invariant Group Values, Difference to Average and Significance

Group	Value	Difference	SE	P-value
0	-0.027	0.003	0.010	0.777
1	-0.034	-0.004	0.012	0.730
2	-0.030	0.000	0.010	0.977
3	-0.029	0.001	0.007	0.914

Intercept for NP_WAIS4

Group	Group	Value	Value	Difference	SE	P-value

Approximate Invariance Was Not Found For This Parameter.

Intercept for NP_GPEG_

Group	Group	Value	Value	Difference	SE	P-value
1	0	-0.021	0.248	-0.268	0.051	0.000
2	0	-0.116	0.248	-0.364	0.051	0.000
2	1	-0.116	-0.021	-0.095	0.046	0.040
3	0	-0.203	0.248	-0.451	0.056	0.000
3	1	-0.203	-0.021	-0.183	0.051	0.000
3	2	-0.203	-0.116	-0.087	0.051	0.087

Approximate Measurement Invariance Holds For Groups:
2 3
Weighted Average Value Across Invariant Groups: -0.157
R-square/Explained variance/Invariance index: 0.070

Invariant Group Values, Difference to Average and Significance

Group	Value	Difference	SE	P-value
2	-0.116	0.041	0.024	0.087
3	-0.203	-0.046	0.027	0.087

Intercept for CNP_SPCP

Group	Group	Value	Value	Difference	SE	P-value
1	0	0.001	-0.198	0.199	0.092	0.031
2	0	0.137	-0.198	0.335	0.061	0.000
2	1	0.137	0.001	0.136	0.061	0.026
3	0	0.128	-0.198	0.325	0.063	0.000
3	1	0.128	0.001	0.126	0.071	0.075
3	2	0.128	0.137	-0.009	0.040	0.814

Approximate Measurement Invariance Holds For Groups:
0 1
Weighted Average Value Across Invariant Groups: -0.103
R-square/Explained variance/Invariance index: 0.209

Invariant Group Values, Difference to Average and Significance

Group	Value	Difference	SE	P-value
0	-0.198	-0.095	0.044	0.031
1	0.001	0.104	0.048	0.031

Intercept for CNP_PCET

Group	Group	Value	Value	Difference	SE	P-value

Approximate Invariance Was Not Found For This Parameter.

Intercept for MEANSVT

Group	Group	Value	Value	Difference	SE	P-value
1	0	0.017	0.017	0.000	0.014	0.978
2	0	0.027	0.017	0.010	0.014	0.468
2	1	0.027	0.017	0.010	0.016	0.532
3	0	0.007	0.017	-0.009	0.011	0.389
3	1	0.007	0.017	-0.010	0.012	0.426
3	2	0.007	0.027	-0.019	0.018	0.282

Approximate Measurement Invariance Holds For Groups:
0 1 2 3
Weighted Average Value Across Invariant Groups: 0.017

R-square/Explained variance/Invariance index: 0.975

Invariant Group Values, Difference to Average and Significance

Group	Value	Difference	SE	P-value
0	0.017	-0.001	0.007	0.940
1	0.017	0.000	0.008	0.986
2	0.027	0.010	0.011	0.368
3	0.007	-0.010	0.009	0.257

Intercept for MEANFC

Group	Group	Value	Value	Difference	SE	P-value

Approximate Invariance Was Not Found For This Parameter.

Loadings

Loadings for CNP_SF2B

Group	Group	Value	Value	Difference	SE	P-value
1	0	0.507	0.493	0.015	0.031	0.638
2	0	0.502	0.493	0.009	0.023	0.694
2	1	0.502	0.507	-0.006	0.029	0.844
3	0	0.488	0.493	-0.005	0.017	0.780
3	1	0.488	0.507	-0.020	0.022	0.366
3	2	0.488	0.502	-0.014	0.023	0.547

Approximate Measurement Invariance Holds For Groups:
0 1 2 3
Weighted Average Value Across Invariant Groups: 0.498
R-square/Explained variance/Invariance index: 0.990

Invariant Group Values, Difference to Average and Significance

Group	Value	Difference	SE	P-value
0	0.493	-0.005	0.015	0.733
1	0.507	0.010	0.019	0.601
2	0.502	0.004	0.016	0.796
3	0.488	-0.010	0.010	0.327

Loadings for CNP_SPCP

Group	Group	Value	Value	Difference	SE	P-value
1	0	0.438	0.558	-0.120	0.084	0.155
2	0	0.274	0.558	-0.284	0.069	0.000
2	1	0.274	0.438	-0.164	0.079	0.038
3	0	0.258	0.558	-0.299	0.070	0.000
3	1	0.258	0.438	-0.180	0.080	0.025
3	2	0.258	0.274	-0.016	0.061	0.797

Approximate Measurement Invariance Holds For Groups:
1 2 3
Weighted Average Value Across Invariant Groups: 0.329
R-square/Explained variance/Invariance index: 0.209

Invariant Group Values, Difference to Average and Significance

Group	Value	Difference	SE	P-value
1	0.438	0.109	0.047	0.020
2	0.274	-0.055	0.039	0.161
3	0.258	-0.071	0.042	0.091

Loadings for CNP_PCET

Group	Group	Value	Value	Difference	SE	P-value
1	0	0.456	0.447	0.009	0.058	0.880
2	0	0.485	0.447	0.038	0.067	0.569
2	1	0.485	0.456	0.029	0.070	0.676
3	0	0.627	0.447	0.180	0.091	0.049
3	1	0.627	0.456	0.171	0.094	0.069
3	2	0.627	0.485	0.142	0.091	0.119

Approximate Measurement Invariance Holds For Groups:
0 1 2 3
Weighted Average Value Across Invariant Groups: 0.497
R-square/Explained variance/Invariance index: 0.646

Invariant Group Values, Difference to Average and Significance

Group	Value	Difference	SE	P-value
0	0.447	-0.050	0.038	0.187
1	0.456	-0.041	0.042	0.321
2	0.485	-0.012	0.045	0.789
3	0.627	0.130	0.066	0.050

Loadings

Loadings for CNP_CPW_

Group	Group	Value	Value	Difference	SE	P-value
1	0	0.528	0.512	0.017	0.038	0.661
2	0	0.545	0.512	0.033	0.040	0.413
2	1	0.545	0.528	0.017	0.039	0.668
3	0	0.516	0.512	0.005	0.036	0.899
3	1	0.516	0.528	-0.012	0.036	0.742
3	2	0.516	0.545	-0.028	0.038	0.454

Approximate Measurement Invariance Holds For Groups:
0 1 2 3
Weighted Average Value Across Invariant Groups: 0.525
R-square/Explained variance/Invariance index: 0.927

Invariant Group Values, Difference to Average and Significance

Group	Value	Difference	SE	P-value
0	0.512	-0.013	0.023	0.556
1	0.528	0.003	0.023	0.885
2	0.545	0.020	0.025	0.427
3	0.516	-0.009	0.023	0.705

Loadings for MEANSVT

Group	Group	Value	Value	Difference	SE	P-value
1	0	0.576	0.552	0.024	0.041	0.557
2	0	0.573	0.552	0.021	0.035	0.551
2	1	0.573	0.576	-0.003	0.034	0.926
3	0	0.569	0.552	0.017	0.038	0.660
3	1	0.569	0.576	-0.007	0.035	0.831
3	2	0.569	0.573	-0.004	0.029	0.884

Approximate Measurement Invariance Holds For Groups:
0 1 2 3
Weighted Average Value Across Invariant Groups: 0.567
R-square/Explained variance/Invariance index: 0.973

Invariant Group Values, Difference to Average and Significance

Group	Value	Difference	SE	P-value
0	0.552	-0.015	0.024	0.529
1	0.576	0.009	0.023	0.693
2	0.573	0.006	0.019	0.751
3	0.569	0.002	0.021	0.934

Loadings for MEANFC

Group	Group	Value	Value	Difference	SE	P-value
1	0	0.539	0.644	-0.105	0.061	0.082
2	0	0.499	0.644	-0.145	0.057	0.011
2	1	0.499	0.539	-0.040	0.051	0.430
3	0	0.596	0.644	-0.048	0.056	0.389
3	1	0.596	0.539	0.057	0.052	0.267
3	2	0.596	0.499	0.097	0.049	0.049

Approximate Measurement Invariance Holds For Groups:
0 1 2 3
Weighted Average Value Across Invariant Groups: 0.572
R-square/Explained variance/Invariance index: 0.406

Invariant Group Values, Difference to Average and Significance

Group	Value	Difference	SE	P-value
0	0.644	0.073	0.036	0.042
1	0.539	-0.032	0.033	0.330
2	0.499	-0.072	0.031	0.021
3	0.596	0.025	0.032	0.444

Loadings
Loadings for CNP_PVRT

Group	Group	Value	Value	Difference	SE	P-value
1	0	0.778	0.781	-0.003	0.034	0.920
2	0	0.766	0.781	-0.015	0.033	0.647
2	1	0.766	0.778	-0.012	0.034	0.725
3	0	0.734	0.781	-0.048	0.037	0.196
3	1	0.734	0.778	-0.044	0.037	0.236
3	2	0.734	0.766	-0.032	0.037	0.382

Approximate Measurement Invariance Holds For Groups:
0 1 2 3
Weighted Average Value Across Invariant Groups: 0.766
R-square/Explained variance/Invariance index: 0.385

Invariant Group Values, Difference to Average and Significance

Group	Value	Difference	SE	P-value
0	0.781	0.015	0.020	0.453
1	0.778	0.011	0.021	0.582
2	0.766	0.000	0.021	0.985
3	0.734	-0.033	0.025	0.187

Loadings for CNP_PMAT

Group	Group	Value	Value	Difference	SE	P-value
1	0	0.628	0.625	0.003	0.030	0.919
2	0	0.638	0.625	0.013	0.029	0.648
2	1	0.638	0.628	0.010	0.029	0.725
3	0	0.662	0.625	0.038	0.028	0.174
3	1	0.662	0.628	0.035	0.028	0.213
3	2	0.662	0.638	0.024	0.027	0.356

Approximate Measurement Invariance Holds For Groups:
0 1 2 3
Weighted Average Value Across Invariant Groups: 0.637
R-square/Explained variance/Invariance index: 0.695

Invariant Group Values, Difference to Average and Significance

Group	Value	Difference	SE	P-value
0	0.625	-0.012	0.017	0.484
1	0.628	-0.009	0.018	0.613
2	0.638	0.001	0.017	0.950
3	0.662	0.026	0.017	0.131

Loadings
Loadings for CNP_MEDF

Group	Group	Value	Value	Difference	SE	P-value
1	0	0.399	0.407	-0.008	0.039	0.830
2	0	0.424	0.407	0.016	0.022	0.461
2	1	0.424	0.399	0.025	0.033	0.450

3	0	0.353	0.407	-0.054	0.070	0.440
3	1	0.353	0.399	-0.046	0.071	0.519
3	2	0.353	0.424	-0.070	0.080	0.377

Approximate Measurement Invariance Holds For Groups:
0 1 2 3
Weighted Average Value Across Invariant Groups: 0.398
R-square/Explained variance/Invariance index: 0.549

Invariant Group Values, Difference to Average and Significance

Group	Value	Difference	SE	P-value
0	0.407	0.010	0.021	0.641
1	0.399	0.001	0.026	0.954
2	0.424	0.026	0.026	0.308
3	0.353	-0.044	0.056	0.428

Loadings for CNP_ER40

Group	Group	Value	Value	Difference	SE	P-value
1	0	0.283	0.275	0.007	0.036	0.836
2	0	0.217	0.275	-0.058	0.065	0.369
2	1	0.217	0.283	-0.066	0.062	0.290
3	0	0.305	0.275	0.029	0.029	0.312
3	1	0.305	0.283	0.022	0.017	0.197
3	2	0.305	0.217	0.088	0.071	0.220

Approximate Measurement Invariance Holds For Groups:
0 1 2 3
Weighted Average Value Across Invariant Groups: 0.270
R-square/Explained variance/Invariance index: 0.741

Invariant Group Values, Difference to Average and Significance

Group	Value	Difference	SE	P-value
0	0.275	0.006	0.024	0.808
1	0.283	0.013	0.020	0.508
2	0.217	-0.052	0.048	0.278
3	0.305	0.035	0.024	0.146

Loadings
Loadings for NP_WRAT4

Group	Group	Value	Value	Difference	SE	P-value
1	0	0.762	0.705	0.057	0.039	0.144
2	0	0.770	0.705	0.065	0.040	0.102
2	1	0.770	0.762	0.008	0.030	0.796
3	0	0.745	0.705	0.040	0.037	0.287
3	1	0.745	0.762	-0.017	0.030	0.564
3	2	0.745	0.770	-0.025	0.034	0.452

Approximate Measurement Invariance Holds For Groups:
0 1 2 3
Weighted Average Value Across Invariant Groups: 0.744
R-square/Explained variance/Invariance index: 0.000

Invariant Group Values, Difference to Average and Significance

Group	Value	Difference	SE	P-value
0	0.705	-0.039	0.025	0.113
1	0.762	0.018	0.019	0.351
2	0.770	0.026	0.021	0.224
3	0.745	0.001	0.021	0.975

Loadings for NP_WRAT4

Group	Group	Value	Value	Difference	SE	P-value
1	0	0.829	0.887	-0.058	0.039	0.136
2	0	0.769	0.887	-0.118	0.040	0.003
2	1	0.769	0.829	-0.060	0.038	0.112

3	0	0.777	0.887	-0.110	0.041	0.007
3	1	0.777	0.829	-0.053	0.038	0.162
3	2	0.777	0.769	0.008	0.034	0.827

Approximate Measurement Invariance Holds For Groups:
0 1 2 3
Weighted Average Value Across Invariant Groups: 0.820
R-square/Explained variance/Invariance index: 0.282

Invariant Group Values, Difference to Average and Significance

Group	Value	Difference	SE	P-value
0	0.887	0.067	0.024	0.005
1	0.829	0.009	0.023	0.679
2	0.769	-0.051	0.023	0.028
3	0.777	-0.043	0.024	0.070

Loadings for CNP_PVOC

Group	Group	Value	Value	Difference	SE	P-value
1	0	0.751	0.768	-0.017	0.025	0.497
2	0	0.782	0.768	0.013	0.021	0.528
2	1	0.782	0.751	0.031	0.032	0.340
3	0	0.803	0.768	0.035	0.027	0.207
3	1	0.803	0.751	0.052	0.036	0.149
3	2	0.803	0.782	0.021	0.031	0.501

Approximate Measurement Invariance Holds For Groups:
0 1 2 3
Weighted Average Value Across Invariant Groups: 0.774
R-square/Explained variance/Invariance index: 0.547

Invariant Group Values, Difference to Average and Significance

Group	Value	Difference	SE	P-value
0	0.768	-0.006	0.011	0.582
1	0.751	-0.023	0.020	0.239
2	0.782	0.007	0.017	0.674
3	0.803	0.028	0.022	0.192

Loadings
Loadings for NP_WAIS4

Group	Group	Value	Value	Difference	SE	P-value
1	0	2.538	-0.837	3.375	0.538	0.000
2	0	2.743	-0.837	3.580	0.375	0.000
2	1	2.743	2.538	0.205	0.181	0.256
3	0	2.692	-0.837	3.529	0.457	0.000
3	1	2.692	2.538	0.154	0.221	0.486
3	2	2.692	2.743	-0.051	0.216	0.812

Approximate Measurement Invariance Holds For Groups:
1 2 3
Weighted Average Value Across Invariant Groups: 2.653
R-square/Explained variance/Invariance index: 0.000

Invariant Group Values, Difference to Average and Significance

Group	Value	Difference	SE	P-value
1	2.538	-0.115	0.108	0.284
2	2.743	0.090	0.109	0.408
3	2.692	0.039	0.139	0.782

Loadings for NP_GPEG_

Group	Group	Value	Value	Difference	SE	P-value
1	0	-1.473	0.614	-2.086	0.196	0.000
2	0	-1.337	0.614	-1.951	0.332	0.000
2	1	-1.337	-1.473	0.136	0.155	0.383
3	0	-1.378	0.614	-1.991	0.278	0.000

```
    3         1     -1.378     -1.473      0.095      0.143      0.507
    3         2     -1.378     -1.337     -0.040      0.178      0.820
 Approximate Measurement Invariance Holds For Groups:
 1 2 3
 Weighted Average Value Across Invariant Groups:      -1.399
 R-square/Explained variance/Invariance index:       0.000

 Invariant Group Values, Difference to Average and Significance
 Group      Value Difference         SE    P-value
    1     -1.473     -0.074      0.077      0.335
    2     -1.337      0.061      0.099      0.536
    3     -1.378      0.021      0.098      0.832

 Average Invariance index:       0.483
```

GUR+ Model: Invariance by Sex
Sex (0: Female; 1: Male)

```
APPROXIMATE MEASUREMENT INVARIANCE (NONINVARIANCE) FOR GROUPS

 Intercepts/Thresholds
   CNP_SF2B    0 1
   CNP_CPW_    0 1
   CNP_PVRT    (0) (1)
   CNP_PMAT    0 1
   CNP_MEDF    0 1
   CNP_ER40    0 1
   NP_WRAT4    (0) (1)
   NP_WRAT4    0 1
   CNP_PVOC    0 1
   NP_WAIS4    (0) (1)
   NP_GPEG_    0 1
   CNP_SPCP    0 1
   CNP_PCET    (0) (1)
   MEANSVT     0 1
   MEANFC      (0) (1)

 Loadings for EXFUNC
   CNP_SF2B    0 1
   CNP_SPCP    0 1
   CNP_PCET    0 1

 Loadings for EPISMEM
   CNP_CPW_    0 1
   MEANSVT     0 1
   MEANFC      0 1

 Loadings for CMPLXCOG
   CNP_PVRT    0 1
   CNP_PMAT    0 1

 Loadings for SOCCOG
   CNP_MEDF    0 1
   CNP_ER40    0 1

 Loadings for GENAB
   NP_WRAT4    0 1
   NP_WRAT4    0 1
   CNP_PVOC    0 1

 Loadings for MOTORSP
   NP_WAIS4    (0) (1)
```

```
   NP_GPEG_    (0) (1)


FACTOR MEAN COMPARISON AT THE 5% SIGNIFICANCE LEVEL IN DESCENDING ORDER

 Results for Factor EXFUNC

          Latent    Group      Factor
 Ranking    Class    Value        Mean     Groups With Significantly Smaller Factor Mean
     1        2         1       0.041
     2        1         0       0.000

 Results for Factor EPISMEM

          Latent    Group      Factor
 Ranking    Class    Value        Mean     Groups With Significantly Smaller Factor Mean
     1        2         1       0.019
     2        1         0       0.000

 Results for Factor CMPLXCOG

          Latent    Group      Factor
 Ranking    Class    Value        Mean     Groups With Significantly Smaller Factor Mean
     1        2         1       0.110
     2        1         0       0.000

 Results for Factor SOCCOG

          Latent    Group      Factor
 Ranking    Class    Value        Mean     Groups With Significantly Smaller Factor Mean
     1        1         0       0.000
     2        2         1      -0.269

 Results for Factor GENAB

          Latent    Group      Factor
 Ranking    Class    Value        Mean     Groups With Significantly Smaller Factor Mean
     1        2         1       0.146    0
     2        1         0       0.000

 Results for Factor MOTORSP

          Latent    Group      Factor
 Ranking    Class    Value        Mean     Groups With Significantly Smaller Factor Mean
     1        1         0       0.000
     2        2         1      -0.030

ALIGNMENT OUTPUT

INVARIANCE ANALYSIS

 Intercepts/Thresholds
 Intercept for CNP_SF2B
 Group     Group       Value      Value     Difference  SE       P-value
     1         0     -0.025     -0.130      0.105      0.088      0.230
 Approximate Measurement Invariance Holds For Groups:
 0 1
 Weighted Average Value Across Invariant Groups:      -0.079
 R-square/Explained variance/Invariance index:       0.294

 Invariant Group Values, Difference to Average and Significance
 Group      Value Difference         SE    P-value
     0     -0.130     -0.052      0.043      0.230
```

```
    1     -0.025      0.054      0.045      0.230

Intercept for CNP_CPW_
Group     Group      Value      Value     Difference  SE       P-value
    1         0      0.041      0.280     -0.240      0.076      0.002
Approximate Measurement Invariance Holds For Groups:
0 1
Weighted Average Value Across Invariant Groups:       0.163
R-square/Explained variance/Invariance index:       0.000

Invariant Group Values, Difference to Average and Significance
Group      Value Difference         SE    P-value
    0      0.280      0.118      0.037      0.002
    1      0.041     -0.122      0.038      0.002

Intercept for CNP_PVRT
Group     Group      Value      Value     Difference  SE       P-value
Approximate Invariance Was Not Found For This Parameter.

Intercept for CNP_PMAT
Group     Group      Value      Value     Difference  SE       P-value
    1         0     -0.035     -0.138      0.102      0.135      0.447
Approximate Measurement Invariance Holds For Groups:
0 1
Weighted Average Value Across Invariant Groups:      -0.088
R-square/Explained variance/Invariance index:       0.655

Invariant Group Values, Difference to Average and Significance
Group      Value Difference         SE    P-value
    0     -0.138     -0.050      0.066      0.447
    1     -0.035      0.052      0.068      0.447

Intercept for CNP_MEDF
Group     Group      Value      Value     Difference  SE       P-value
    1         0      0.128      0.108      0.020      0.019      0.291
Approximate Measurement Invariance Holds For Groups:
0 1
Weighted Average Value Across Invariant Groups:       0.118
R-square/Explained variance/Invariance index:       1.000

Invariant Group Values, Difference to Average and Significance
Group      Value Difference         SE    P-value
    0      0.108     -0.010      0.010      0.291
    1      0.128      0.010      0.010      0.291

Intercept for CNP_ER40
Group     Group      Value      Value     Difference  SE       P-value
    1         0     -0.239     -0.159     -0.080      0.106      0.447
Approximate Measurement Invariance Holds For Groups:
0 1
Weighted Average Value Across Invariant Groups:      -0.198
R-square/Explained variance/Invariance index:       0.740

Invariant Group Values, Difference to Average and Significance
Group      Value Difference         SE    P-value
    0     -0.159      0.039      0.052      0.447
    1     -0.239     -0.041      0.054      0.447

Intercept for NP_WRAT4
Group     Group      Value      Value     Difference  SE       P-value
Approximate Invariance Was Not Found For This Parameter.

Intercept for NP_WRAT4
```

```
Group     Group      Value      Value     Difference  SE       P-value
    1         0     -0.106     -0.138      0.032      0.038      0.400
Approximate Measurement Invariance Holds For Groups:
0 1
Weighted Average Value Across Invariant Groups:      -0.123
R-square/Explained variance/Invariance index:       0.954

Invariant Group Values, Difference to Average and Significance
Group      Value Difference         SE    P-value
    0     -0.138     -0.016      0.018      0.400
    1     -0.106      0.016      0.019      0.400

Intercept for CNP_PVOC
Group     Group      Value      Value     Difference  SE       P-value
    1         0     -0.065     -0.082      0.016      0.028      0.557
Approximate Measurement Invariance Holds For Groups:
0 1
Weighted Average Value Across Invariant Groups:      -0.074
R-square/Explained variance/Invariance index:       0.980

Invariant Group Values, Difference to Average and Significance
Group      Value Difference         SE    P-value
    0     -0.082     -0.008      0.014      0.557
    1     -0.065      0.008      0.014      0.557

Intercept for NP_WAIS4
Group     Group      Value      Value     Difference  SE       P-value
Approximate Invariance Was Not Found For This Parameter.

Intercept for NP_GPEG_
Group     Group      Value      Value     Difference  SE       P-value
    1         0      0.259      0.239      0.020      0.043      0.645
Approximate Measurement Invariance Holds For Groups:
0 1
Weighted Average Value Across Invariant Groups:       0.249
R-square/Explained variance/Invariance index:       0.000

Invariant Group Values, Difference to Average and Significance
Group      Value Difference         SE    P-value
    0      0.239     -0.010      0.021      0.645
    1      0.259      0.010      0.022      0.645

Intercept for CNP_SPCP
Group     Group      Value      Value     Difference  SE       P-value
    1         0     -0.215     -0.192     -0.022      0.035      0.518
Approximate Measurement Invariance Holds For Groups:
0 1
Weighted Average Value Across Invariant Groups:      -0.203
R-square/Explained variance/Invariance index:       0.000

Invariant Group Values, Difference to Average and Significance
Group      Value Difference         SE    P-value
    0     -0.192      0.011      0.017      0.518
    1     -0.215     -0.011      0.018      0.518

Intercept for CNP_PCET
Group     Group      Value      Value     Difference  SE       P-value
Approximate Invariance Was Not Found For This Parameter.

Intercept for MEANSVT
Group     Group      Value      Value     Difference  SE       P-value
    1         0      0.054     -0.017      0.070      0.064      0.270
Approximate Measurement Invariance Holds For Groups:
```

```
0 1
Weighted Average Value Across Invariant Groups:       0.018
R-square/Explained variance/Invariance index:       0.256

Invariant Group Values, Difference to Average and Significance
Group      Value Difference         SE    P-value
    0     -0.017     -0.035      0.031      0.270
    1      0.054      0.036      0.032      0.270

Intercept for MEANFC
Group     Group      Value      Value     Difference  SE       P-value
Approximate Invariance Was Not Found For This Parameter.



Loadings
Loadings for CNP_SF2B
Group     Group      Value      Value     Difference  SE       P-value
    1         0      0.490      0.475      0.015      0.061      0.803
Approximate Measurement Invariance Holds For Groups:
0 1
Weighted Average Value Across Invariant Groups:       0.483
R-square/Explained variance/Invariance index:       0.915

Invariant Group Values, Difference to Average and Significance
Group      Value Difference         SE    P-value
    0      0.475     -0.007      0.030      0.803
    1      0.490      0.008      0.031      0.803

Loadings for CNP_SPCP
Group     Group      Value      Value     Difference  SE       P-value
    1         0      0.546      0.536      0.011      0.059      0.859
Approximate Measurement Invariance Holds For Groups:
0 1
Weighted Average Value Across Invariant Groups:       0.541
R-square/Explained variance/Invariance index:       0.958

Invariant Group Values, Difference to Average and Significance
Group      Value Difference         SE    P-value
    0      0.536     -0.005      0.029      0.859
    1      0.546      0.005      0.030      0.859

Loadings for CNP_PCET
Group     Group      Value      Value     Difference  SE       P-value
    1         0      0.344      0.515     -0.170      0.087      0.049
Approximate Measurement Invariance Holds For Groups:
0 1
Weighted Average Value Across Invariant Groups:       0.431
R-square/Explained variance/Invariance index:       0.000

Invariant Group Values, Difference to Average and Significance
Group      Value Difference         SE    P-value
    0      0.515      0.084      0.042      0.049
    1      0.344     -0.087      0.044      0.049

Loadings
Loadings for CNP_CPW_
Group     Group      Value      Value     Difference  SE       P-value
    1         0      0.529      0.532     -0.003      0.057      0.959
Approximate Measurement Invariance Holds For Groups:
0 1
Weighted Average Value Across Invariant Groups:       0.531
R-square/Explained variance/Invariance index:       0.995
```

Invariant Group Values, Difference to Average and Significance

Group	Value	Difference	SE	P-value
0	0.532	0.001	0.028	0.959
1	0.529	-0.001	0.029	0.959

Loadings for MEANSVT

Group	Group	Value	Value	Difference	SE	P-value
1	0	0.595	0.567	0.028	0.074	0.708

Approximate Measurement Invariance Holds For Groups:
0 1
Weighted Average Value Across Invariant Groups: 0.580
R-square/Explained variance/Invariance index: 0.000

Invariant Group Values, Difference to Average and Significance

Group	Value	Difference	SE	P-value
0	0.567	-0.014	0.036	0.708
1	0.595	0.014	0.038	0.708

Loadings for MEANFC

Group	Group	Value	Value	Difference	SE	P-value
1	0	0.650	0.676	-0.026	0.076	0.728

Approximate Measurement Invariance Holds For Groups:
0 1
Weighted Average Value Across Invariant Groups: 0.663
R-square/Explained variance/Invariance index: 0.874

Invariant Group Values, Difference to Average and Significance

Group	Value	Difference	SE	P-value
0	0.676	0.013	0.037	0.728
1	0.650	-0.013	0.039	0.728

Loadings

Loadings for CNP_PVRT

Group	Group	Value	Value	Difference	SE	P-value
1	0	0.815	0.809	0.006	0.053	0.914

Approximate Measurement Invariance Holds For Groups:
0 1
Weighted Average Value Across Invariant Groups: 0.812
R-square/Explained variance/Invariance index: 0.991

Invariant Group Values, Difference to Average and Significance

Group	Value	Difference	SE	P-value
0	0.809	-0.003	0.026	0.914
1	0.815	0.003	0.027	0.914

Loadings for CNP_PMAT

Group	Group	Value	Value	Difference	SE	P-value
1	0	0.647	0.652	-0.005	0.043	0.914

Approximate Measurement Invariance Holds For Groups:
0 1
Weighted Average Value Across Invariant Groups: 0.650
R-square/Explained variance/Invariance index: 0.993

Invariant Group Values, Difference to Average and Significance

Group	Value	Difference	SE	P-value
0	0.652	0.002	0.021	0.914
1	0.647	-0.002	0.022	0.914

Loadings for CNP_MEDF

Group	Group	Value	Value	Difference	SE	P-value

```
    1         0      0.489      0.348      0.141      0.189      0.456
 Approximate Measurement Invariance Holds For Groups:
 0 1
 Weighted Average Value Across Invariant Groups:       0.417
 R-square/Explained variance/Invariance index:       0.000

 Invariant Group Values, Difference to Average and Significance
 Group      Value Difference         SE    P-value
    0      0.348     -0.069      0.093      0.456
    1      0.489      0.072      0.096      0.456

 Loadings for CNP_ER40
 Group     Group      Value      Value     Difference  SE       P-value
    1         0      0.268      0.287     -0.019      0.014      0.185
 Approximate Measurement Invariance Holds For Groups:
 0 1
 Weighted Average Value Across Invariant Groups:       0.277
 R-square/Explained variance/Invariance index:       0.841

 Invariant Group Values, Difference to Average and Significance
 Group      Value Difference         SE    P-value
    0      0.287      0.009      0.007      0.185
    1      0.268     -0.010      0.007      0.185

Loadings for NP_WRAT4
 Group     Group      Value      Value     Difference  SE       P-value
    1         0      0.581      0.757     -0.175      0.068      0.010
 Approximate Measurement Invariance Holds For Groups:
 0 1
 Weighted Average Value Across Invariant Groups:       0.670
 R-square/Explained variance/Invariance index:       0.000

 Invariant Group Values, Difference to Average and Significance
 Group      Value Difference         SE    P-value
    0      0.757      0.086      0.033      0.010
    1      0.581     -0.089      0.035      0.010

 Loadings for NP_WRAT4
 Group     Group      Value      Value     Difference  SE       P-value
    1         0      0.857      0.829      0.028      0.040      0.477
 Approximate Measurement Invariance Holds For Groups:
 0 1
 Weighted Average Value Across Invariant Groups:       0.843
 R-square/Explained variance/Invariance index:       0.940

 Invariant Group Values, Difference to Average and Significance
 Group      Value Difference         SE    P-value
    0      0.829     -0.014      0.020      0.477
    1      0.857      0.014      0.020      0.477

 Loadings for CNP_PVOC
 Group     Group      Value      Value     Difference  SE       P-value
    1         0      0.739      0.720      0.019      0.031      0.528
 Approximate Measurement Invariance Holds For Groups:
 0 1
 Weighted Average Value Across Invariant Groups:       0.729
 R-square/Explained variance/Invariance index:       0.959

 Invariant Group Values, Difference to Average and Significance
 Group      Value Difference         SE    P-value
    0      0.720     -0.010      0.015      0.528
    1      0.739      0.010      0.016      0.528
```

Loadings for NP_WAIS4

Group	Group	Value	Value	Difference	SE	P-value
1	0	0.846	-0.794	1.641	0.112	0.000

Approximate Invariance Was Not Found For This Parameter.

Loadings for NP_GPEG_

Group	Group	Value	Value	Difference	SE	P-value
1	0	-0.576	0.614	-1.190	0.101	0.000

Approximate Invariance Was Not Found For This Parameter.

Average Invariance index: 0.580
